# Multiple roles of PP2A binding motif in hepatitis B virus core linker and PP2A in regulating core phosphorylation state and viral replication

**DOI:** 10.1371/journal.ppat.1009230

**Published:** 2021-01-25

**Authors:** Ji Xi, Laura Luckenbaugh, Jianming Hu

**Affiliations:** Department of Microbiology and Immunology, The Pennsylvania State University College of Medicine, Hershey, Pennsylvania, United States of America; University of California, San Diego, UNITED STATES

## Abstract

Hepatitis B virus (HBV) capsid or core protein (HBc) contains an N-terminal domain (NTD) and a C-terminal domain (CTD) connected by a short linker peptide. HBc plays a critical role in virtually every step of viral replication, which is further modulated by dynamic phosphorylation and dephosphorylation of its CTD. While several cellular kinases have been identified that mediate HBc CTD phosphorylation, there is little information on the cellular phosphatases that mediate CTD dephosphorylation. Herein, a consensus binding motif for the protein phosphatase 2A (PP2A) regulatory subunit B56 was recognized within the HBc linker peptide. Mutations within this motif designed to block or enhance B56 binding showed pleiotropic effects on CTD phosphorylation state as well as on viral RNA packaging, reverse transcription, and virion secretion. Furthermore, linker mutations affected the HBV nuclear episome (the covalently closed circular or CCC DNA) differentially during intracellular amplification vs. infection. The effects of linker mutations on CTD phosphorylation state varied with different phosphorylation sites and were only partially consistent with the linker motif serving to recruit PP2A-B56, specifically, to dephosphorylate CTD, suggesting that multiple phosphatases and/or kinases may be recruited to modulate CTD (de)phosphorylation. Furthermore, pharmacological inhibition of PP2A could decrease HBc CTD dephosphorylation and increase the nuclear HBV episome. These results thus strongly implicate the HBc linker in recruiting PP2A and other host factors to regulate multiple stages of HBV replication.

## Author summary

Hepatitis B virus (HBV) causes acute and chronic viral hepatitis, liver fibrosis, cirrhosis and cancer. The dynamic phosphorylation and dephosphorylation of the viral capsid protein (HBc), which are controlled by host cell protein kinases and phosphatases, play a critical role in regulating multiple stages of HBV replication. While a number of cellular kinases have been identified that mediate HBc phosphorylation, there is little information on cellular phosphatases that mediate its dephosphorylation. Herein we have identified a consensus binding motif in HBc for one of the major cellular phosphatases, the protein phosphatase 2A (PP2A). Genetic analysis of this motif revealed that it played multiple roles in regulating CTD phosphorylation state, as well as viral RNA packaging, reverse transcription, virion secretion, and formation of the nuclear HBV episome responsible for viral persistence. Furthermore, pharmacological inhibition of PP2A decreased HBc dephosphorylation and increased the nuclear episome, further supporting a role of PP2A in HBc dephosphorylation and HBV persistence. These results thus suggest that HBc recruits PP2A, among other host factors, to regulate HBc phosphorylation and dephosphorylation dynamics and HBV replication and persistence.

## Introduction

Hepatitis B virus (HBV) is the prototype member of the *Hepadnaviridae* family and the cause of acute and chronic hepatitis B, liver fibrosis, cirrhosis and hepatocellular carcinoma (HCC) [1–3]. HBV genome is a small (3.2 kb), partially double-stranded (DS), relaxed circular (RC) DNA, in which neither strand is covalently closed [4]. In complete HBV virions, RC DNA is enclosed in an icosahedral capsid formed by the HBV core protein (HBc), which is in turn enclosed by the viral envelope containing three surface proteins (large, middle, small or L, M, S). During HBV infection, RC DNA is converted to a covalently closed circular (CCC) DNA, the key viral DNA species serving as the sole transcription template able to produce all viral RNAs essential for viral replication, including the pregenomic RNA (pgRNA), the precursor to the progeny RC DNA [5–7].

In infected cells, RC DNA synthesis begins with incorporation of pgRNA, together with the viral reverse transcriptase (RT) protein, into an immature nucleocapsid (NC) [5,8]. pgRNA then undergoes reverse transcription catalyzed by the RT protein [9], which firstly converts it to a single-stranded (SS) (of minus strand polarity) DNA and subsequently to RC DNA. Only RC DNA-containing (mature) NCs can be enveloped by the viral surface proteins to secrete extracellularly as complete virions [10]. RC DNA inside mature NCs, in addition to being secreted in complete virions, can also be imported into the nucleus via the so-called intracellular recycling pathway [11–15]. In addition to complete virions, empty HBV capsids, without any RNA or DNA packaged, are also formed inside cell, enveloped by the surface proteins, and secreted as empty virions at high levels [10,16,17].

HBc is a 21 kDa protein that plays a key role in multiple steps of HBV replication [18]. The N-terminal domain (NTD, 1–140) of HBc forms the capsid shell [19,20] and also plays a role in pgRNA packaging, reverse transcription, virion secretion, and CCC DNA formation [21–28]. The arginine-rich C-terminal domain (CTD, 150–183) of HBc contains three major SP phosphorylation sites (S155, S162, and S170) and four additional S/T phosphorylation sites (T160, S168, S176 and S178) and is involved in regulating capsid assembly, pgRNA packaging, reverse transcription, and RC DNA nuclear import [21,22,29–39]. We have recently shown that the short (9-residues, 141–149) linker peptide between NTD and CTD, instead of serving merely as a spacer with no specific functions, also plays a critical role in multiple stages of viral replication [40].

The HBc CTD undergoes dynamic phosphorylation and dephosphorylation that regulates multiple stages of HBV replication. CTD is hyperphosphorylated in HBc dimers and initial dephosphorylation may promote pgRNA-packaging [29,41,42]. During reverse transcription and NC maturation, the doubling of interior NC negative charge, as a result of conversion of (SS) pgRNA to (DS) RC DNA, may be counter balanced by CTD dephosphorylation, so as to stabilize mature NCs [31,33,43]. Thus, CTD in mature NCs is extensively or completely dephosphorylated [33,44]. On the other hand, CTD dephosphorylation is not required for envelope interactions to secrete either DNA-containing or empty virions [44]. To allow RC DNA release for CCC DNA formation in the nucleus, mature NCs formed inside infected cells or from incoming virions have to disassemble, at least partially, in an ill-understood process termed uncoating [7,27,28,45]. Our recent evidence suggests that uncoating requires rephosphorylation of CTD, and potentially de novo phosphorylation of NTD, of mature NCs [28].

**Fig 1:**
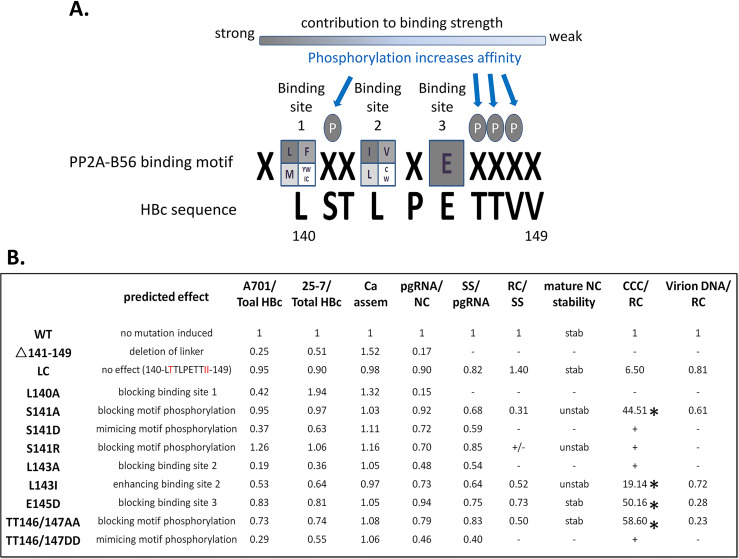
Summary of the effects of HBc linker mutants on different steps of viral replication. **A.** Alignment of HBc linker sequence (`141–149), including the last residue of NTD (140), with the consensus PP2A-B56 binding motif. **B.** Mutations in the B56 binding motif and predicted effects on B56 binding, and observed effects of the mutations on HBc CTD dephosphorylation at two distinct mAb epitopes—A701 (T160/S162) and 25–7 (S178)—both normalized to total HBc levels, capsid assembly (Ca assem), pgRNA packaging (pgRNA/NC), SS DNA synthesis (SS/pgRNA), RC DNA synthesis (RC/SS), mature NC stability, CCC DNA synthesis (CCC/RC), and DNA virion secretion (virion DNA/RC), with the level for each parameter from the WT HBc set to 1.0. N/A, not applicable due to the lack of detection of RC DNA; -/+, very weak signal detected; -, below the limit of detection; unstab, unstable NC; stab, stable NC as WT; *, CCC DNA decreased during infection. +, CCC DNA generated but no detectable core RC DNA, so CCC DNA/RC DNA normalization was impossible. The red font in the mutant LC indicates the amino acid change from the WT sequence (S141T and VV148/149II).

A number of host cell kinases have been implicated in phosphorylating HBc, in particular CTD, including the cyclin-dependent kinase 2 (CDK2), which is also shown to represent the major activity of the so-called “endogenous kinase” packaged into HBV capsids [28,46], protein kinase A and C (PKA and PKC) [47,48], serine-arginine protein kinase 1 and 2 (SRPK1 and SRPK2) [49,50], and Polo-like kinase 1 [51]. However, there is very little information about which phosphatases are responsible for HBc/CTD dephosphorylation. Protein phosphatase 2A (PP2A) constitutes the most abundant source of phosphatase activity in human cells and regulates numerous cellular processes and signaling pathways. PP2A is a heterotrimeric holoenzyme composed of a catalytic subunit (PP2A-C), a scaffolding subunit (PP2A-A), and one of a large array of regulatory B subunits, which provide substrate specificity [52]. The largest regulatory B subunit family-B56, which comprises five human isoforms (α, β, γ, δ, and ε), can bind to a consensus sequence motif on the interacting proteins, LxxI/LxE, in which L_1_, I/L_4_ and E_6_ constitute the crucial binding sites (Fig 1A) [53–55]. Furthermore, phosphorylation at position 2 within the motif and immediately downstream serves to further enhance B56 binding.

We have recently shown that substitutions within the linker peptide can affect HBc CTD phosphorylation state, which implicates an important role of the linker sequence in regulating CTD phosphorylation [40]. We report here the identification of a PP2A-B56 consensus binding motif within the HBc linker and our mutagenesis efforts to determine the role of this motif in regulating HBc phosphorylation and HBV replication. Using specific pharmacological inhibitors, we have also explored the role of PP2A in HBc dephosphorylation and in HBV replication. Our results support an important role of the HBc linker peptide in recruiting PP2A and possibly other cellular phosphatase/kinases to regulate the CTD phosphorylation state and consequently, control multiple stages of HBV replication. A specific role of PP2A in dephosphorylating HBc during NC maturation and uncoating is also implicated.

## Materials and methods

### Cell cultures

HepG2 cells were cultured in Dulbecco modified Eagle F-12 medium supplemented with 10% fetal bovine serum and 50 ug/ml of penicillin-streptomycin. HepAD38 cells, which are stably transfected with a cDNA copy of HBV wild-type pregenomic RNA and in which viral replication is under the control of a tetracyclin-repressible promoter [56], were cultured in Dulbecco modified Eagle F-12 medium supplemented with 10% fetal bovine serum, 50 ug/ml of penicillin-streptomycin, 400 μg/ml of G418 and 5 μg/ml of tetracyclin. Induction of viral replication in HepAD38 cells is achieved by with removing tetracyclin from the culture medium.

### Plasmids

pCI-HBc WT, pCI-HBcΔ141–149, pCI-HBc-LC, pCI-HBc-3A, and pCI-HBc-3E for expression of the WT and mutant HBc proteins have been previously described [29,40]. pCI-HBc linker mutants L140A, S141A, S141D, S141R, L143A, L143I, E145D, TT146/147AA, and TT146/147DD were constructed by in-Fusion mutagenesis. The HBV replicon plasmid pCIΔA-HBV-HBc-WT was constructed by subcloning the entire HBV insert together with the linked human cytomegalovirus (HCMV) promoter sequences from pCMVHBV [57,58] to the pCIΔA vector and has been described recently [27,28]. pCIΔA-HBV-HBc-C^-^, was constructed by substitution of core ORF 4^th^ codon (GAC) to a stop codon (TAG), which is capable of supporting viral replication upon complementation with HBc. pCIΔA-HBV-HBc-L^-^, was constructed by substitution of the L ORF start codon (ATG) to ACG. pCIΔA-HBV-HBc-TT146/147AA was constructed by introducing the TT146/147AA linker double substitutions into pCIΔA-HBV-HBc-WT and expresses the mutated HBV pgRNA encoding the same HBc linker mutation as in pCI-HBc-TT146/147AA. pCIΔA-HBV-HBc-TT146/147AA-L(-) was constructed from pCIΔA-HBV-HBc-WT-L(-) by introducing the same linker double substitutions as in pCI-HBc-TT146/147AA.

### Antibodies

The mAb, clone 1D8, specific for the NTD of HBc (epitope approximately from 78–82) was a gift from Peter Revill (Victorian Infectious Diseases Reference Laboratory, Australia). The rabbit polyclonal antibody against HBc was purchased from Dako. A mouse monoclonal antibody (mAb), clone T2221, against the HBc NTD was purchased from Tokyo Future Style (Cat no. 2AHC24) [40]. The HBc CTD-specific mAb A701, which specifically recognizes dephosphorylated T160/S162, and mAb 25–7, which recognizes dephosphorylated HBc CTD, have been described before [29,40,44]. The rabbit mAb specific for the pS-Q motif, Phospho-ATM/ATR Substrate Motif (S*Q), was purchased from Cell Signaling Technology (CST #9607). The rabbit anti-HBs polyclonal antibody was purchased from Virostat.

### Transient transfection

HepG2 cells were seeded in 60-mm dishes and transfected with 4 μg (total) of plasmid using X-tremeGENE HP DNA Transfection Reagent (Roche). Cells and culture supernatant were harvested on day seven post-transfection.

### SDS-PAGE and western blot analysis

Cells were lysed with the NP40 lysis buffer (50 mM Tris-HCl [pH 8.0], 1 mM EDTA, 1% NP-40 containing the protease inhibitor cocktail (Roche) and a mix of phosphatase inhibitor cocktail 2 and 3 (Sigma)) and the cytoplasm lysate was collected after a brief spin to remove nuclei [58,59]. HBc proteins in the cytoplasmic lysate were resolved by sodium dodecyl sulfate-polyacrylamide gel electrophoresis (SDS-PAGE) and transferred to polyvinylidene difluoride (PVDF) membrane for detection by the indicated antibodies.

### Phos-tag gel electrophoresis

Resolution of different phosphor-isomers of HBc in cytoplasmic lysates by Phos-tag gel electrophoresis was conducted as described [28,43,49,60], with minor modifications. Briefly, cytoplasmic lysates containing HBc proteins were boiled in SDS sample buffer containing 1 mM MnCl2 and 10% β-mercaptoethanol for 10 min and then resolved on a Phos-tag SDS-polyacrylamide gel (10%-acrylamide:bis-acrylamide -ratio 37.5:1, 100 μM Phos-tag acrylamide (Wako Pure Chemical Corporation), 200 μM MnCl_2_). After electrophoresis, the gel was soaked in the transfer buffer containing 10 mM EDTA with gentle shaking for 10 min each time to chelate the Mn^2+^ ion left in the gel which could impact the transfer efficiency, with one to three buffer changes, before transfer to PVDF membrane.

### Capsid assembly and RNA packaging

To determine the levels of assembled capsids and the efficiency of pgRNA packaged inside capsids, cytoplasmic lysates were resolved on 1% native agarose gels (native agarose gel electrophoresis or NAGE). Upon transfer of resolved capsids onto nitrocellulose membrane, the packaged pgRNA was detected using a ^32^P-labeled anti-sense RNA probe as described previously [44,58]. Subsequently, capsids on the same membrane was detected by using the indicated HBc antibodies.

### Isolation and analysis of viral DNA

For isolation of HBV core DNA, cytoplasmic lysates were digested with 0.6 mg/ml of proteinase K (PK)and 0.5% sodium dodecyl sulfate (SDS) at 37°C for 1 h. HBV core DNA was purified by phenol-chloroform extraction and ethanol precipitation [58,59]. For analysis of capsid stability, the cytoplasmic lysates were digested with TURBO DNase (0.2 U/μl) for 30 min. Subsequently, EDTA (20 mM final concentration) was added to terminate the digestion. The lysates were then further digested with SDS (0.5%)/PK (0.6 mg/ml) and DNA was purified by phenol-chloroform ethanol precipitation. Where indicated, *Dpn* I (NEB) digestion, instead of TURBO DNase, was used to remove the transfected plasmid after DNA purification [27]. Protein-free (PF) DNA (including CCC DNA) was isolated by a modified Hirt extraction procedure as described previously [27,61]. Purified DNAs were digested with Dpn I (NEB) to remove the transfected plasmids. To remove the nicked viral DNA and plasmid DNA signals, the *Dpn* I-digested DNA was further digested with Exonuclease I (Exo I) and Exonuclease III (Exo III) (NEB) as described [61]. Briefly, 20 ul PF DNA (from a total of 200 ul per 60 mm dish of cells) was digested with 20 units of Exo I and 25 units of Exo III in the 1x Cutsmart buffer (NEB) at 37°C for 2 hrs. Viral DNA was resolved on a 1.2% agarose gel and detected by standard Southern blot analysis using a ^32^P-labeled HBV DNA probe. Where indicated, core DNA was also analyzed by releasing it from NCs using SDS-proteinase K digestion and resolving the released DNA directly on an agarose gel without purification, as described [28,45].

### Virion secretion assay

Cell culture supernatant was concentrated (by 50 times) by polyethylene glycol (PEG) precipitation as described [17,40]. Concentrated viral particles were digested with *DNase* I to remove plasmid DNAs and resolved on a 1% native agarose gel. Viral DNA was detected using a ^32^P-labeled HBV DNA probe following transfer to nitrocellulose membrane. Subsequently, the same membrane was probed with the indicated core- or surface-specific antibody to detect the core or surface protein.

### Analysis of HBc dephosphorylation using rabbit reticulocyte lysate (RRL) in vitro translation system

A TnT-coupled rabbit reticulocyte lysate (RRL) (Promega) in vitro translation and capsid assembly system was used to express the WT or mutant HBc proteins, as described previously [29,62]. In vitro translated proteins were incubated in the 1X NEB Buffer 3 containing the 1X EDTA-free Protease Inhibitor (cOmplete) and 1X RNasin Plus Ribonuclease Inhibitor (Promega, N2611) for 16 hrs at 37°C to allow HBc dephosphorylation by endogenous RRL phosphatases. Alkaline Phosphatase, Calf Intestinal (CIAP) (NEB, M0290S) (10U) was added to some reactions as a positive control for dephosphorylation. To prevent dephosphorylation, the 1X Phosphatase inhibitor cocktail 2&3 (Sigma, P5726&P0044), or specific inhibitors of cellular phosphatases, including okadaic acid (Sigma, O9381) (1 nM, 10 nM, 100 nM) and fostriecin (10 nM, 20 nM,40 nM, 80 nM) (Tocris, 1840) was added following translation and before the dephosphorylation reaction. HBc proteins were then resolved by NAGE, SDS-PAGE, or Phos-tag SDS-PAGE and detected by western blot analysis using the indicated HBc antibodies.

### HBV infection

HBV infection in HepG2-NTCP was carried out as previously described [28,61], with slight modifications. Briefly, HepG2-NTCP cells were plated in collagen I-coated 35-mm tissue culture dishes. When the cells reached 50% confluence, they were pretreated with 2% DMSO for 8 days to arrest cell growth. The cells were then infected by using the HBV inoculum harvested from HepAD38 cells, at a multiplicity of infection (MOI) of ca. 100–200 genome equivalent (GE)/cell in the presence of 2% dimethyl sulfoxide (DMSO), 4% polyethylene glycol (PEG) 8000. The next day, the viral inoculum was removed and fresh medium containing 2% DMSO was added. Where indicated, the PP2A inhibitor fostriecin (20 μM, 40 μM, 80 μM) was added following the removal of the inoculum and maintained until the cells were harvested three days post-infection. HBV PF DNA was extracted and analyzed by Southern blot analysis as described above.

### Phosphatase inhibitor treatment of HepAD38 cells

Tetracyclin was withdrawn from the culture medium for two days to initiate HBV expression. Phosphonoformic acid (PFA, 2 mM) was then added for four days to arrest HBV DNA synthesis at the single-stranded DNA stage [28,63,64]. Subsequently, PFA was removed and the PP2A inhibitor fostriecin (20 μM, 40 μM, 60 μM) was added. One day later, the cells were harvested for analysis of HBc proteins and HBV core DNA and CCC DNA as described above.

### Quantification and statistical analysis

All experiments were repeated at least 3 times. DNA signals from Southern blot analysis were detected by phosphor imaging using the Typhoon 9500 imager (GE Healthcare Life Sciences) and quantified using Quantity ONE (Bio-Rad) or using the Sapphire Biomolecular Imager (Azure Biosystems). Protein signals from western blot analysis were detected and quantified using the Bio-Rad Chemi-Doc (ImageLab) system.

## Results

### Identification of a consensus PP2A/B56 binding motif in the HBc linker

We have recently shown that the HBc linker peptide plays a critical role in multiple steps of HBV replication, including the modulation of CTD phosphorylation state [40]. In our attempts to understand how the linker may exert its multiple effects, we identified a consensus binding motif for the PP2A regulatory subunit B56, which, as recently characterized, serves to recruit B56-containing PP2A holoenzymes to their specific substrates for dephosphorylation [53–55]. The entire binding motif, LxxI/LxE, is contained within the linker plus the very last residue of the NTD (Fig 1A). Thus, the highly conserved HBc sequence, _140_**L**ST**L**P**E**TTVV_149_, conforms perfectly to the B56 consensus binding motif (critical binding residues in bold), including the perfect matches of HBc L140 at position 1 and E145 at position 6 of the motif, as well as L143 at position 4, which is occupied by Leu only less frequently than by Ile. To ascertain the role of this motif in HBV replication including the regulation of HBc phosphorylation, we targeted the three residues within the motif, L_1_, I/L_4_ and E_6_, critical for B56 binding for mutagenesis, to disrupt or enhance B56 binding (Fig 1B). Furthermore, we also targeted the potential sites of phosphorylation within and immediately downstream the motif to either block or mimic phosphorylation, as phosphorylation at these positions have been shown to further enhance B56 binding (Fig 1B).

### B56 consensus site mutations in the HBc linker affected the CTD phosphorylation state in a site-specific manner

**Fig 2:**
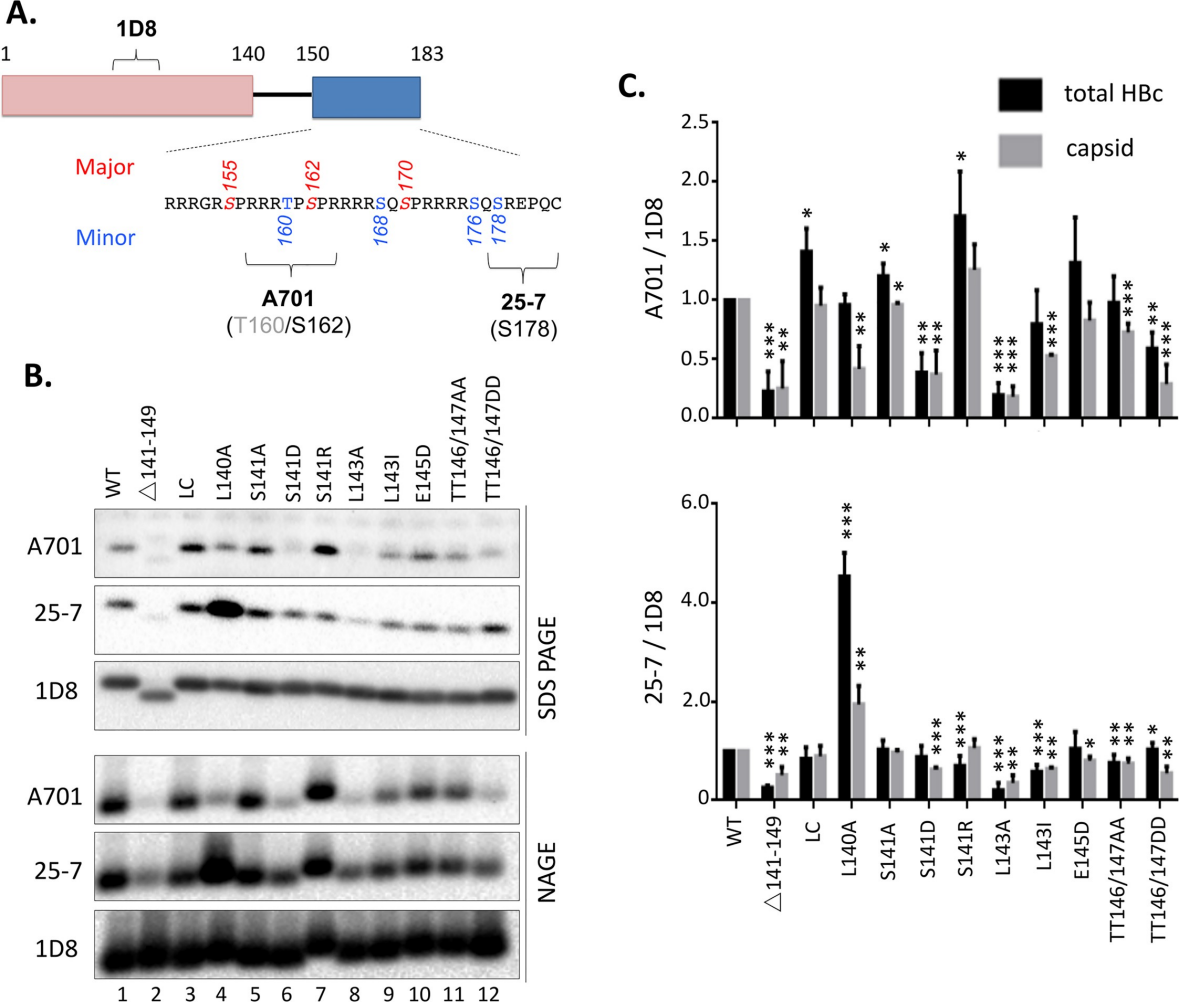
Effects of HBc linker mutations on CTD state of phosphorylation. **A.** Schematic of HBc domain structure and mAb epitope locations. The NTD and CTD are depicted as pink and blue boxes, with the linker peptide in between as a thin line. Epitopes recognized by the HBc-specific mAbs: 1D8 for NTD (ca. 70–80), independent of CTD state of phosphorylation; A701 for unphosphorylated S162/T160; and 25–7 for unphosphorylated S178. **B.** HepG2 cells were transfected with indicated HBc expression constructs and were harvested seven days post-transfection. Cytoplasmic lysates from the transfected cells were resolved by SDS-PAGE (top) or NAGE (bottom) and detected by western blot analysis, using the NTD-specific mAb 1D8 (for total HBc proteins) and two non-phosphorylated CTD-specific mAbs, A701 and 25–7. **C.** The CTD dephosphorylation signals from total HBc (quantified following SDS-PAGE; black bars) or capsids (quantified following NAGE; gray bars) were normalized to total HBc or capsid signal (A701/1D8, top; 25-7/1D8, bottom). The normalized A701 or 25–7 signals of the linker mutants are shown relative to that of the WT HBc, which was set to 1.00. *, P<0.05; **, P<0.01;***, P<0.001.

To determine if the linker mutations could affect HBc dephosphorylation, we took advantage of two mAbs that selectively recognize dephosphorylated HBc CTD at two different locations: A701 and 25–7. mAb A701 recognizes an epitope between 156–163, selective for dephosphorylated S162—one of the major CTD sites of phosphorylation, and likely also for dephosphorylated T160—one of the minor phosphorylation sites (Fig 2A) [44]. mAb 25–7 is selective for a non-phosphorylated epitope within 164–182 of CTD, which contains four sites of phosphorylation (S168, S170, S176, and S178) (Fig 2A) [29,40,44]. Here, we have further mapped the 25–7 epitope to a region containing S178, i.e., it selectively recognized non-phosphorylated S178 (Fig 2A). From the previous results [29] and again in S1A and S1B Fig here, it was clear that S170 is not part of the epitope since the mutant HBc 3A or 3E (in which the three major sites of phosphorylation including S170 are substituted with Ala or Glu respectively) was recognized well by mAb 25–7. We employed here the pSQ specific mAb to determine if mAb 25–7 recognized either SQ motif in CTD (i.e., S168 and S176). The specificity of the pSQ mAb for phosphorylated HBc was first verified by its failure to detect (non-phosphorylated) HBc produced in E. coli and ability to detect (phosphorylated) HBc produced in HepG2 cells (S1C Fig). Furthermore, the failure of the pSQ mAb to detect either 7A or 7E produced in HepG2 cells was consistent with its recognition of one or both of the pSQ sites in the CTD, which, together with all other CTD phosphorylation sites, were substituted with A or E in 7A or 7E respectively, resulting in the loss of the pSQ mAb epitope (S1D Fig). Interestingly, 3A expressed both cell lines showed dramatically enhanced reactivity with the pSQ specific mAb compared to WT, indicating enhanced phosphorylation at either or both of the S-Q sites (S1B and S1D Fig). If mAb 25–7 recognizes either one of these two SQ sites, it would react with 3A much weaker than WT as the 25–7 epitope is non-phosphorylated, or equally as the WT if 3A only enhanced phosphorylation at one of the two SQ sites and the SQ site recognized by 25–7 and the pSQ mAb is different. However, we observed that 25–7 recognized 3A even better than the WT HBc, thus indicating that 25–7 didn't recognize either S-Q site in CTD (S1B Fig). Therefore, we were able to locate the mAb 25–7 epitope to S178 (non-phosphorylated) and demonstrate that the 3A mutant decreased phosphorylation at S178 such that it reacted better with mAb 25–7.

Following resolution of HBc by SDS-PAGE (Fig 2B /top/) or NAGE (Fig 2B /bottom/), total HBc levels were measured by using the NTD-specific mAb 1D8, which were used to normalize the dephosphorylated HBc detected by mAb A701 or 25–7 (Fig 2C). Both the total HBc protein (subunit) (SDS-PAGE) and capsid (NAGE) levels from all the linker mutants were similar to WT. Regarding the levels of CTD dephosphorylation, the LC mutant—with no change on the B56 binding motif, showed no obvious effect on CTD dephosphorylation at either CTD epitope (Fig 2B /lane 3/); In contrast, the linker deletion mutant strongly decreased CTD dephosphorylation at both epitopes of CTD (Fig 2B /lane 2/), in agreement with our previous results on these two mutants [40]. Interestingly, L140A, predicted to impair B56 binding, reduced dephosphorylation at the A701 epitope but dramatically enhanced dephosphorylation at the 25–7 epitope (Fig 2B /lane 4/). L143A, also predicted to impair B56 binding, reduced dephosphorylation at both the A701 and 25–7 epitopes (Fig 2B /lane 8/), while L143I, predicted to maintain B56 binding, did not significantly affect dephosphorylation at either CTD epitope (Fig 2B /lane 9/). However, E145D, predicted to inhibit B56 binding, didn’t reduce significantly CTD dephosphorylation at either epitope (Fig 2B/lane 10/), contrary to our prediction.

Interestingly, substitutions at the three potential linker phosphorylation sites (S141, T146, and T147) seemed to affect CTD phosphorylation in a manner that suggested linker phosphorylation could enhance CTD phosphorylation. Thus, S141D and TT146/147DD, mimicking linker phosphorylation, decreased dephosphorylation (or enhanced phosphorylation) at both CTD epitopes (Fig 2B /lane 6 and 12/). We also attempted to verify directly the anticipated increase in CTD phosphorylation levels using a mAb (14–2) that is selective for phosphorylated CTD (epitope between 164–183). However, as we reported recently [28], since the vast majority of HBc expressed in human cells are heavily phosphorylated, it was difficult to detect any significant increase in CTD phosphorylation levels in any of the linker (or other HBc) mutants over the WT HBc. In contrast, S141A and TT146/147AA didn’t significantly affect dephosphorylation at either CTD epitopes (Fig 2B /lane 5 and 11/), and S141R slightly enhanced HBc dephosphorylation at the A701 epitope (Fig 2B/lane 7/).

### Phos-tag SDS-PAGE revealed multiple changes in HBc phosphorylation state caused by linker mutations

**Fig 3:**
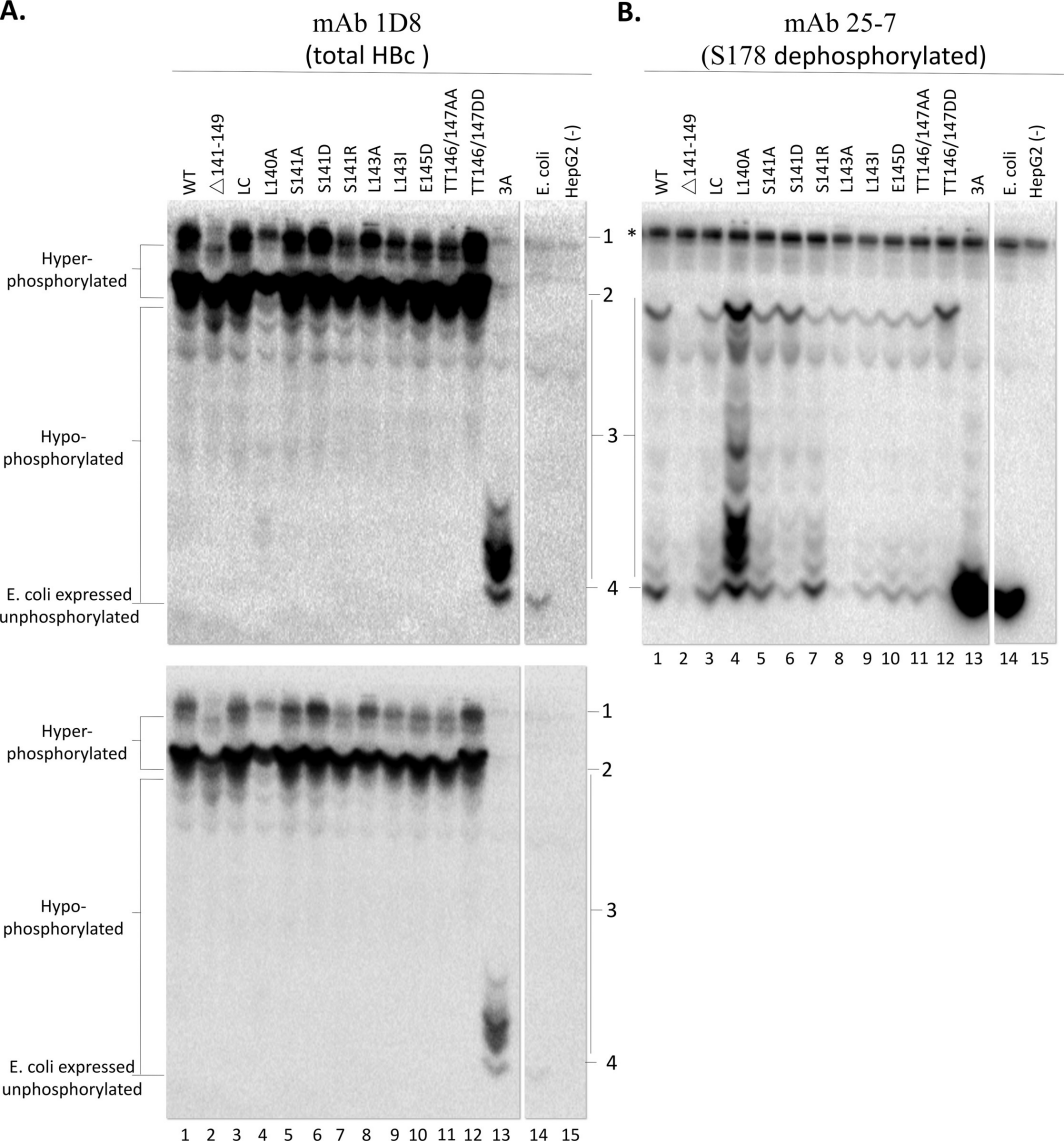
Phos-Tag SDS-PAGE and immunoblot analysis of HBc linker mutants. HepG2 cells were transfected with HBc mutants expression constructs and harvested seven days post-transfection. Cytoplasmic lysates of the transfected cells were resolved by Phos-tag SDS-PAGE and detected by western blot analysis, using the HBc NTD mAb 1D8 for total HBc (**A**) or 25–7 for dephosphorylated S178 (**B**). 1 & 2, hyperphosphorylated HBc species; 3, hypophosphorylated HBc species; 4, non-phosphorylation HBc, *, non-specific signal. The top and bottom panels in A are long and short exposure of the same membrane.

To determine the effects of the linker mutations on HBc phosphorylation state in more details, we turned to Phos-tag gel analysis. Phos-Tag SDS-PAGE can resolve different phosphor-isoforms of a phosphorylated protein based on its degree and possibly, specific site of phosphorylation and we and others have successfully adopted this technology to resolve multiple species of phosphorylated HBc [28,43,49]. As a non-phosphorylated control, we used HBc expressed and purified from E.coli, which is completely non-phosphorylated. In addition, we also used the HBc mutant 3A, in which the three major CTD phosphorylation sites (S155, S162, S170; Fig 2A) are all replaced with Ala, as a hypophosphorylated control [28,49]. As reported by us and others [28,49], the hyperphosphorylated WT HBc expressed in human cells could be resolved into two major species (or more precisely, two major groups of species each with a few closely migrating bands) migrating near the top of the gel, when mAb 1D8 was used to detect all HBc species irrespective of their state of phosphorylation (Fig 3A /lane 1, species 1 and 2/). A few minor, faster-migrating species were also detected, representing minor hypo-phosphorylated WT HBc in human cells (Fig 3A /lane 1, species 3/). Also as expected, HBc purified from E. coli migrated as a single species to the bottom of the gel (Fig 3A /lane 14, species 4/), and 3A from human cells migrated as a series of 5–6 closely-spaced bands, including band 4, near the bottom of the gel (Fig 3A/lane 13/).

When mAb 25–7, which, as described above, recognizes specifically those HBc species dephosphorylated at S178, was used, we detected the WT HBc from E. coli (i.e., nonphosphorylated) as a single strong band migrating as the band detected by mAb 1D8 (Fig 3B /lane 14/), as expected. For the hypo-phosphorylated 3A expressed in human cells, mAb 25–7 detected predominantly the species that co-migrated with the E. coli-derived HBc (Fig 3B /lane 13/). In contrast, the other slower-migrating species of 3A, which were as abundant as or more abundant than the fastest-migrating band based on mAb 1D8, was detected only weakly by mAb 25–7 (Fig 3B /lane 13/), indicating these species were mostly phosphorylated at S178. mAb 25–7 also detected a series of weak bands (labeled as species “3” in Fig 3) migrating above the series of bands in 3A detected by 1D8, indicating these slower migrating species, while representing only minor fractions of total 3A (and thus not even detected by 1D8), could be detected, in a highly sensitive manner, by 25–7 when S178 was unphosphorylated.

For the WT HBc from human cells, neither of the two dominant (hyper-phosphorylated) bands detected by mAb 1D8 near the top of the gel was detected by 25–7 (Fig 3B /lane 1/), indicating that the HBc species represented by these two bands were highly phosphorylated at the 25–7 epitope (and likely also at the other sites), consistent with their severely retarded mobility on the Phos-tag gel. In contrast, mAb 25–7 detected a series of faster-migrating hypo-phosphorylated species (Fig 3B /lane 1, species 3/), including the fastest migrating species that co-migrated with the non-phosphorylated HBc from E. coli (species 4), which were only weakly detected or not detected at all by mAb 1D8 (Fig 3A /lane 1/) due to their low abundance.

When the linker was completely deleted, mAb 1D8 detected the same two slow-migrating, hyper-phosphorylated species (band 1 and 2) as in the WT HBc, although they migrated slightly faster due to their slightly smaller size (Fig 3A /lane 2/). However, mAb 25–7 detected very little signals in the middle and bottom part of the gel representing hypo-phosphorylated and non-phosphorylated species (Fig 3B /lane 2, band 3 and 4/), indicating decreased CTD dephosphorylation at the 25–7 epitope, consistent with the results from the regular SDS-PAGE above (Fig 2). The LC mutant, which is not predicted to change the B56 binding motif, showed nearly an identical spectrum of phosphor-species as the WT, when detected by either mAb 1D8 or 25–7 (Fig 3A and 3B /lane 3/), indicating this mutant had no obvious effect on HBc phosphorylation state, again consistent with the results from the regular SDS-PAGE/ western blot analysis above.

The most striking result from the Phos-tag gel analysis was that L140A displayed multiple bands in the middle of the gel representing hypophosphorylated species (species or group 3) as detected by mAB 25–7 (Fig 3B /ane 4/). These bands in L140A were detected much more strongly by 25–7 than in the WT and additionally, novel 25–7 reactive species, absent from the WT, also appeared to be detected, especially at the top half of species 3 (Fig 3B /lane 4/). Moreover, novel bands of L140A within species 3 could also be detected now by 1D8 (Fig 3A /lane 4/), which were undetectable in the WT HBc (Fig 3A /lane 1/), indicating these dephosphorylated L140A species comprised a significant fraction of the total L140A such that they could be detected by the mAb that is non-selective to hypo- or non-phosphorylated species. These results were thus fully consistent with the strong 25–7 signal of L140A detected by regular SDS-PAGE analysis above (Fig 2) but further revealed that this single, relatively conservative, change at the end of the HBc NTD dramatically altered the HBc phosphorylation state resulting in multiple heterogenous species dephosphorylated at S178.

L143A showed decreased 25–7 signals in species 3 (hypophosphorylated) and species 4 (non-phosphorylated) (Fig 3B /lane 8/), consistent with impaired dephosphorylation as revealed by the SDS-PAGE/western blot analysis above (Fig 2). On the other hand, the phosphor-mimetic S141D decreased the signal of the hypophosphorylated species and non-phosphorylated species as detected by mAb 25–7 (Fig 3B /lane 6, bottom portion of species 3 and species 4/), accompanied by an enhanced signal of the hyperphosphorylated species as detected by mAb 1D8 (Fig 3A /lane 6, species 1/). These results thus indicated that S141D enhanced S178 phosphorylation and/or reduced its dephosphorylation, consistent with the results from the regular SDS-PAGE/western blot analysis above. Interestingly, the other phosphor-mimetic linker mutant, TT146/147DD, further slowed the migration of the hyperphosphorylated species (species 1 and 2) (Fig 3A /lane 12/), as detected by mAb 1D8, suggesting enhanced phosphorylation. Furthermore, the same mutant showed a depletion of the faster migrating species (species 3 and 4) representing hypophosphorylated and non-phosphorylated species (Fig 3B /lane 12/) as detected by mAb 25–7. Thus, both of the phosphor-mimetic mutants in the linker increased HBc phosphorylation and/or decreased its dephosphorylation.

The effect of TT146/147DD on the HBc phosphorylation state could be revealed by the Phos-tag gel analysis more clearly than the regular SDS-PAGE analysis above (Fig 2), highlighting the utility of Phos-tag gels in assessing the HBc phosphorylation state. Similarly, the Phos-tag gels were able to reveal additional effects of other linker mutants on HBc phosphorylation state that were not apparent by regular SDS-PAGE. Thus, whereas Δ141–149 and L143A showed similar degrees of dephosphorylation on both the A701 (T160/S162) and 25–7 (S178) epitopes based on regular SDS-PAGE (Fig 2B /lane 2 and 8/ and 2C), clear differences in HBc phosphorylation state were revealed by the Phos-tag gels between these two mutants (Fig 3 /lane 2 and 8/). When total HBc was detected using mAb 1D8, the level of species 1 (hyperphosphorylated) was lower in Δ141–149 than in L143A. Similarly, when mAb 25–7 was used to detect S178-dephopshorylated HBc, the top most band within species 3, representing relatively high degree of HBc phosphorylation, was clearly lower in Δ141–149 than in L143A. These results from the Phos-tag gels thus all indicated that the phosphorylation level of Δ141–149 was lower than L143A. Also, even though dephosphorylation at the A701 and 25–7 epitopes between S141D and L143I was similar based on regular SDS-PAGE (Fig 2B /lane 6 and 9/ and 2C), the Phos-tag gel analysis again revealed differences in phosphorylation between these two mutants (Fig 3 /lane 6 and 9/). From the result of total HBc analysis (based on mAb 1D8), species 1 (hyperphosphorylated) was stronger in S141D than in L143I. Based on mAb 25–7, S141D showed multiple hypophosphorylated bands in the top portion of region 3, which were weaker or absent in L143I. Both of these results indicated that S141D were phosphorylated more heavily than L143I.

The linker mutants S141A, S141R, E145D and TT146/147AA showed little to no effects on HBc phosphorylation as compared with the WT, based on the Phos-tag gel analysis and detection with either mAb 1D8 or 25–7 (Fig 3 /lane 5, 7, 10,11/), consistent with the regular SDS-PAGE analysis above.

### Effects of B56 consensus site mutations in the HBc linker on capsid assembly and pgRNA packaging

**Fig 4:**
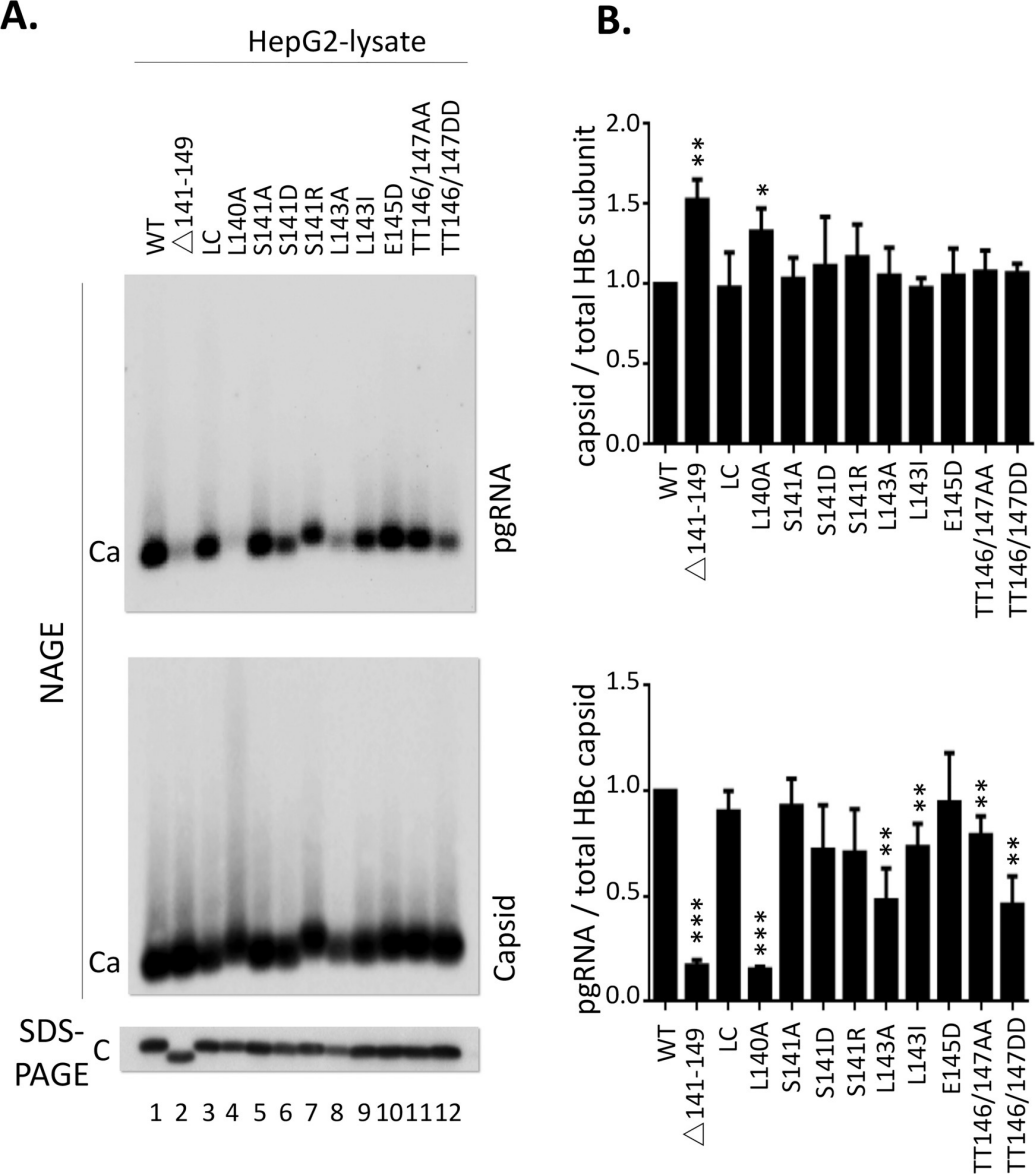
Effect of linker mutations on capsid assembly and pgRNA packaging. HepG2 were co-transfected with the indicated HBc expression constructs and the HBV genomic construct defective in HBc expression and harvested seven days post-transfection. **A.** Cytoplasmic lysates from the transfected cells were resolved by NAGE and transferred to nitrocellulose membrane. The anti-sense HBV RNA probe and the mAb 1D8 were used to detect packaged pgRNA and assembled capsids respectively (top panel). Total HBc levels were measured, following SDS-PAGE and transfer to PVDF membrane, using mAb 1D8 against HBc NTD (bottom panel). Ca, capsid; C, HBc monomer. **B.** Quantitative results from multiple experiments shown in **A.** Capsid assembly efficiency was determined by normalizing the levels of capsids to those of total HBc protein, and RNA packaging efficiency by normalizing the levels of RNA packaging to those of capsids, with the efficiency from WT set to 1.0. *, P<0.05; **, P<0.01;***, P<0.001.

We then tested the effects of the linker mutations on the ability of HBc to assemble capsids and package pgRNA. An HBc expression construct for either the WT or mutant HBc protein, was co-transfected into the human hepatoma cells (HepG2) along with an HBV replicon construct defective in HBc expression. The abilities of the WT or mutant HBc proteins to support various steps of viral replication in this trans-complementation assay could then be determined. Consistent with our recent report showing that the deletion of the entire linker does affect block capsid assembly in human cells [40], none of the linker mutants here impaired capsid assembly as determined by NAGE and western blotting (Fig 4A /NAGE/ and 4B /top/) or HBc expression as determined by SDS-PAGE and western blotting (Fig 4A /bottom/). In addition, consistent with our previous report for a role of the linker in supporting pgRNA packaging, in a sequence-independent manner [40], none of the linker mutants here, except L140A, affected pgRNA packaging significantly (Fig 4A /NAGE/ and 4B /bottom panel/). The linker deletion mutant (Δ141–149), which blocked pgRNA packaging, and a conservative linker substitution mutant (LC: _140_LTTLPETTII_149_), which is not predicted to affect the B56 binding motif and is fully functional in pgRNA packaging, both from our previous study [40], were included here as controls (Fig 4A /lane 2 and 3/). The one linker mutant, L140A, which was predicted to block B56 binding (Fig 4B), decreased pgRNA packaging dramatically (by ca. 4-fold) (Fig 4A /lane 4/ and 4B /bottom panel/), to the same extent as the linker deletion. Two other mutants, L143A and TT146/147DD, modestly reduced pgRNA packaging by ca. 2-fold (Fig 4A /lane 8 and 12, NAGE/ and 4B bottom).

### Effects of B56 consensus site mutations in the HBc linker on viral DNA synthesis

**Fig 5:**
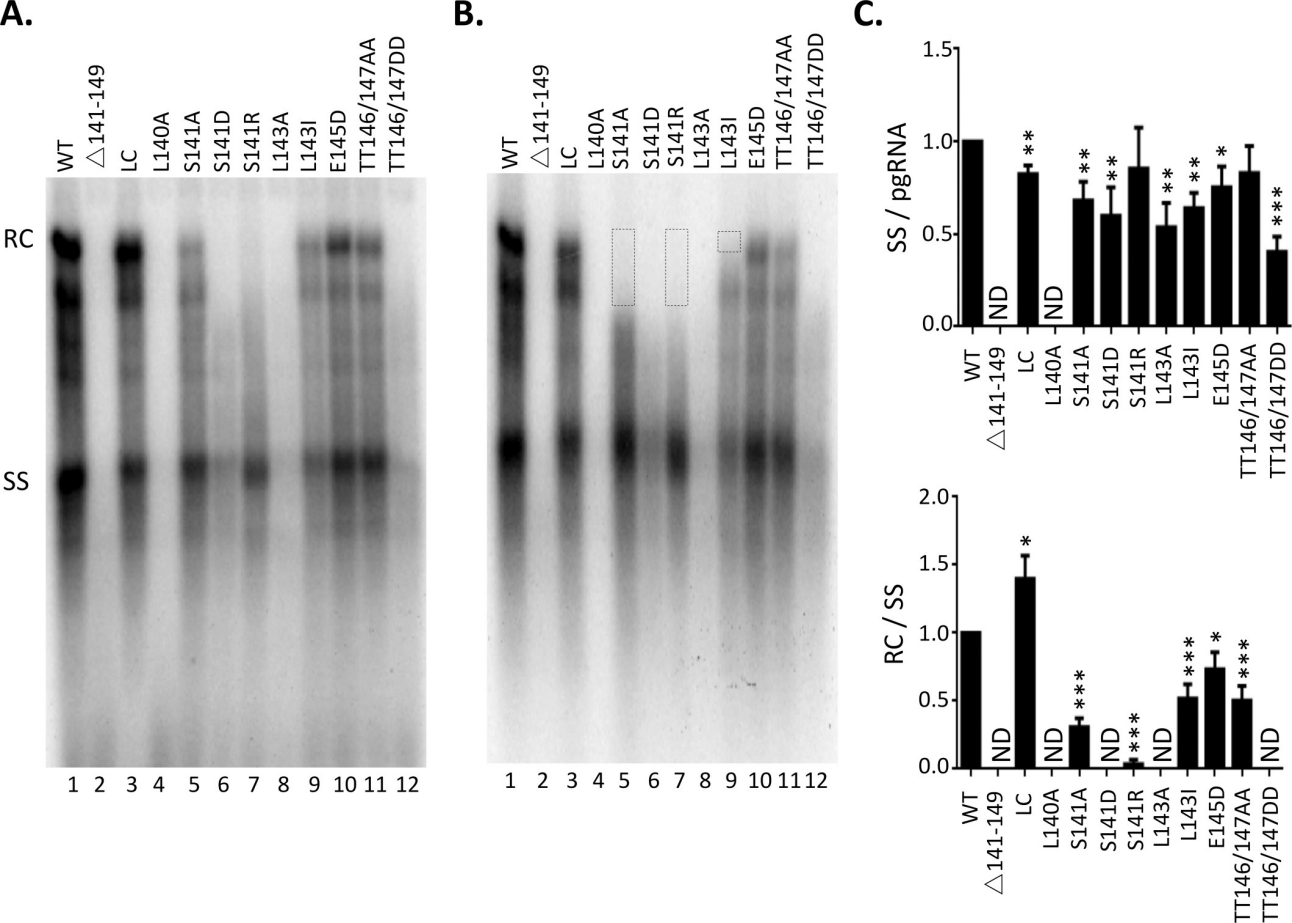
Effect of linker mutations on core DNA. HepG2 were co-transfected with indicated HBc expression constructs and the HBV genomic construct defective in HBc expression and were harvested seven days post-transfection. HBV NC-associated DNA (core DNA) was extracted from cytoplasmic lysate without (**A**) or with TURBO DNase digestion (**B**), and detected by Southern blot analysis. Input plasmid DNA (but not viral replicative DNA) was removed with Dpn I before Southern blot analysis in **A**. The viral DNA signals (RC and immature DS DNA) digested by the nuclease were marked by the dotted boxes (**B**). RC, RC DNA; SS, SS DNA. **C.** Quantitative results from multiple experiments shown in **A.** SS DNA synthesis efficiency was determined by normalizing the levels of SS DNA to the pgRNA signals in Fig 4. RC DNA synthesis efficiency was determined by normalizing the levels of RC DNA to the SS DNA signals. The efficiencies from WT was set to 1.0. *, P<0.05; **, P<0.01;***, P<0.001.

To analyze viral DNA synthesis, we conducted Southern blot analysis of HBV DNA extracted with two alternative NC DNA extraction methods, i.e., with (Fig 5B) or without (Fig 5A) pretreatment of the cytoplasmic lysate with DNase, which is intended to degrade plasmid DNA background but can also degrade viral NC DNA if NCs are unable to protect their DNA content [27,45]. A comparison of the results using both methods can assess not only the overall levels of viral DNA synthesis but also the integrity/stability of viral NCs harboring different viral DNA species. Consistent with the strong defect of L140A in pgRNA packaging, little viral DNA synthesis could be detected in this mutant with either method of DNA extraction (Fig 5A and 5B /lane 4/ and 5C), similar to the effects of the linker deletion mutant (Fig 5A and 5B /lane 26 and 5C) we reported recently [40]. The conservative LC mutant showed little effect on viral DNA synthesis (Fig 5A and 5B /lane 3/ and 5C), as reported before [40].Two linker mutants, E145D and TT146/147AA, also showed little to mild effect (less than 2-fold reduction) on viral DNA synthesis (Fig 5A and 5B /lane 10 and 11/ and 5C).

The other linker mutants showed strong effects on various steps of viral DNA synthesis. Without prior DNase treatment, S141A and L143I showed low levels of mature RC DNA (Fig 5A /lane 5 and 9/), and S141R showed barely detectable RC DNA signal (Fig 5A /lane 7/). The other linker mutants, S141D, L143A, and TT146/147DD, did not show any detectable mature RC DNA but contained variable levels of immature DS DNA intermediates running as a smear between the SS DNA and RC DNA (Fig 5A /lane 6, 8 and 12/), suggesting these mutants were able to initiate plus strand DNA synthesis but deficient in extensive plus strand elongation. With DNase I pretreatment, even S141A, S141R, and L143I now showed no detectable RC DNA, indicating that the low levels of RC DNA synthesized by these mutants could not be protected by their mature NCs (Fig 5B /lane 5, 7 and 9/). In contrast to RC DNA, all the mutants showed detectable SS DNA (Fig 5), except the linker deletion and L140A mutant, which showed little SS DNA as expected from their severe defect in pgRNA packaging, and the S141D and L143I mutants, which showed significantly reduced SS DNA. Also, SS DNA was not significantly affected by the DNase pretreatment, indicating the defect in those linker mutants to protect their DNA was specific to the mature NCs (containing RC DNA). Interestingly, some immature DS DNA, running just below the RC DNA, was also selectively eliminated by the DNase digestion from S141A and S141R (Fig 5B /lane 5 and 7, boxed region/), whereas the same DNA region in L143I, in which RC DNA was eliminated by DNase digestion as in S141A and S141R, was not affected. These results thus suggested that S141A and S141R, but not L143I, also affected the integrity of the NCs containing immature DS DNA though not those containing SS DNA.

### Effects of B56 consensus site mutations in the HBc linker on virion secretion

**Fig 6:**
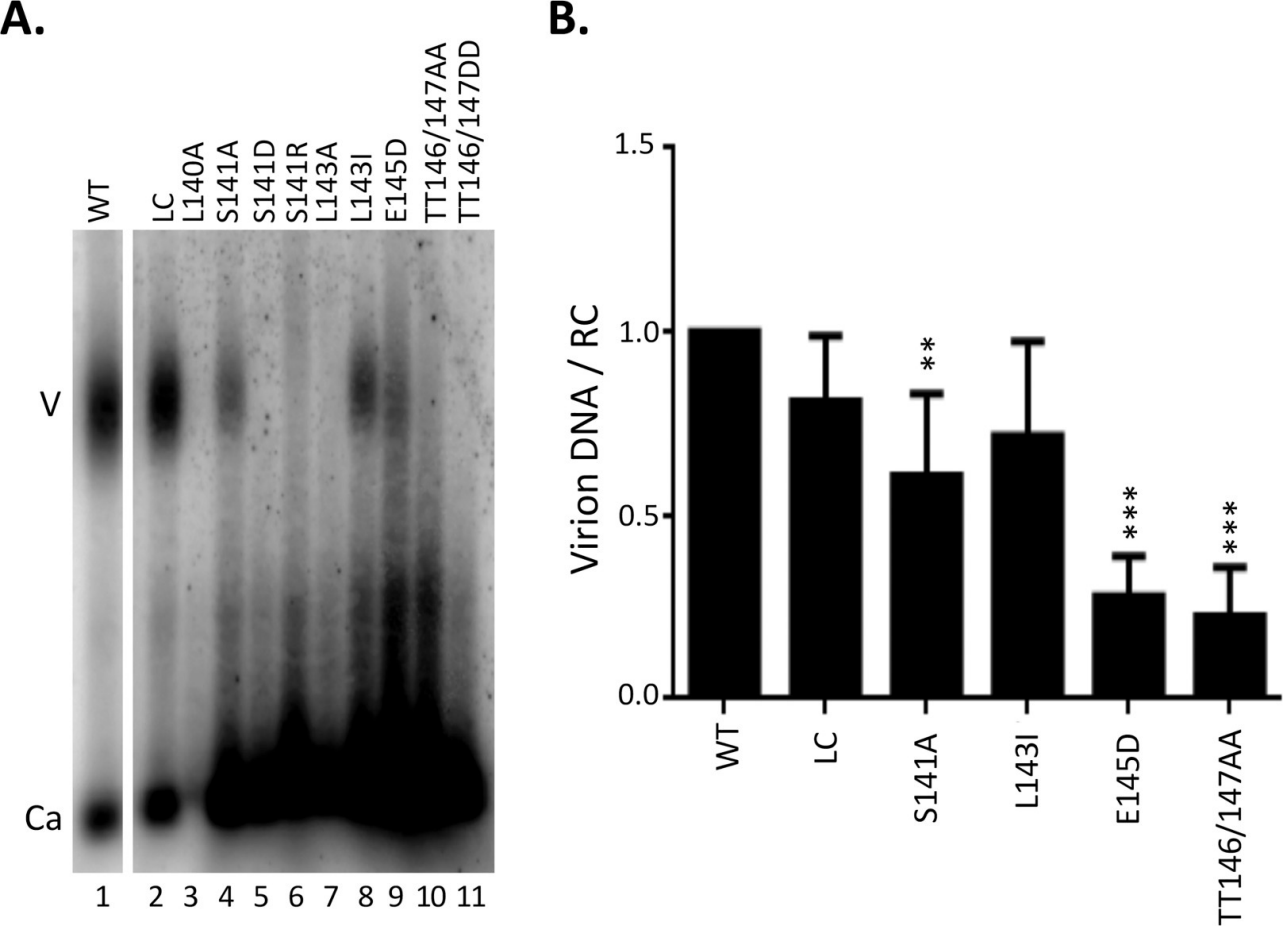
Effect of linker mutations on virion secretion. HepG2 were co-transfected with indicated HBc expression constructs and the HBV genomic construct defective in HBc expression and the culture supernatant was collected seven days post-transfection. **A.** Concentrated culture supernatant was resolved by agarose gel electrophoresis and transferred to nitrocellulose membrane. HBV DNA in virions and naked capsids were detected by using the HBV DNA probe. **B.** DNA virion secretion efficiency was determined by normalizing levels of virion DNA to cytoplasmic RC DNA levels (as shown in Fig 5). The efficiency from WT was set to 1.0. V, virion; Ca, capsid. **, P<0.01;***, P<0.001.

Viral particles released into the culture supernatant of transfected cells were analyzed by NAGE followed by Southern blotting and detection of viral DNA (Fig 6A). Naked capsids were released from all mutants as well as WT, proportional to the intracellular NC-associated DNA levels shown in Fig 5A, indicating no effect by any linker mutants on naked capsid release. On the other hand, only S141A, L143I, and E145D secreted some complete virions (Fig 6A /lane 4, 8 and 9/), and the double mutant (TT146/147AA) showed barely detectable levels of DNA virion secretion (Fig 6A /lane 10/). By normalizing the virion DNA signals with those of the intracellular RC DNA (without DNase pretreatment, Fig 5A), we found that DNA virion secretion efficiency of S141A and L143I, as well as the previously described LC (Fig 6A /lane 2/) [40] was similar to the WT (Fig 6B). In contrast, E145D and TT146/147AA, despite showing strong cytoplasmic RC DNA levels (Fig 5A), showed only low levels of DNA virion secretion, being ca. 4-fold lower than the WT (Fig 6B). These results thus indicated that secretion of complete virions could be influenced by the linker sequence. The effect on virion DNA secretion by the other mutants (L140A, S141D, S141R, L143A, TT146/147DD) couldn’t be assessed here (Fig 6A /lane 3, 5–7 and 11/) since they didn’t show any detectable levels of intracellular RC DNA as shown above (Fig 5A /lane 4, 6–8 and 12/), which is a prerequisite for DNA virion secretion.

### Effects of B56 consensus site mutations in the HBc linker on CCC DNA formation via intracellular amplification

**Fig 7:**
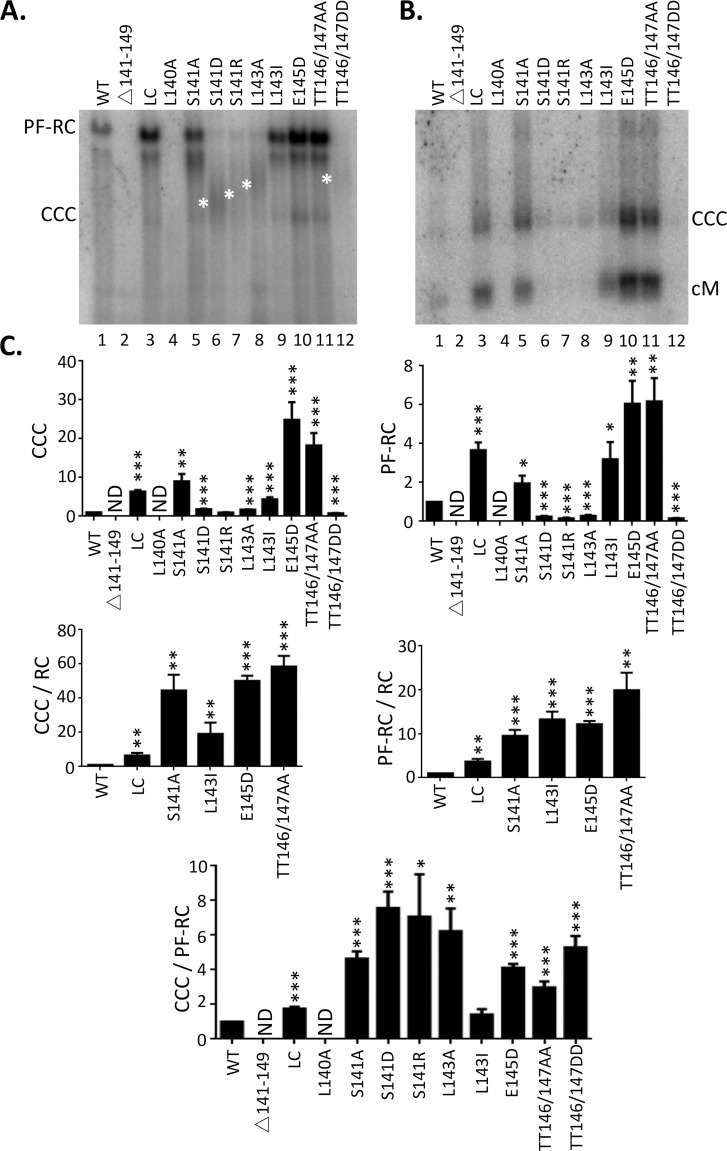
Effects of linker mutations on CCC DNA formation. HepG2 were co-transfected with indicated HBc expression constructs and the HBV genomic construct defective in HBc expression and HBV PF DNA was extracted from the transfected cells seven days after transfection. The extracted DNA was digested with Dpn I to degrade input plasmids (**A**), or Dpn I plus the exonuclease I and III to removal all DNA except closed circular DNA (**B**), before agarose gel electrophoresis and Southern blot analysis. Novel PF DNA smears detected from certain mutants are marked with the white asterisks to the left of the relevant lanes (S141D, S141R, L143A, TT146/147DD). PF-RC, PF-RC DNA; CCC, CCC DNA; cM, closed minus strand DNA. **C.** CCC DNA and PF-RC DNA signals of each mutant were compared with WT (top two panels). CCC DNA and PF-RC DNA are normalized to core RC DNA (middle two panels), and CCC DNA is normalized PF-RC DNA (bottom). All values from the WT were set to 1.0. *, P<0.05; **, P<0.01;***, P<0.001.

Southern blot analysis of PF-DNA extracted from the transfected cells, with or without exonuclease pretreatment to remove all HBV DNA species except covalently closed circles [61], showed multiple effects of the linker mutants on CCC DNA, PF-RC DNA, and other PF DNA species. Except for the linker deletion and L140A mutant, which blocked pgRNA packaging and reverse transcription and thus showed no PF DNA (Fig 7A and 7B /lane 2 and 4/), those mutants that showed similar (LC) or reduced (but detectable) RC DNA (L141A, L143I, E145D, TT146/146AA) all showed significantly increased levels of PF-RC DNA and CCC DNA compared to the WT (Fig 7A and 7B /lane 3, 5, 9, 10 and 11/), indicating these mutants increased the formation of CCC DNA and PF-RC DNA (Fig 7C). Most interestingly, even those mutants (S141D, S141R, L143A, TT146/147/DD) that showed little to no cytoplasmic RC DNA signals still showed CCC DNA levels comparable to the WT (Fig 7B /lane 6, 7, 8 and 12/); On the other hand, the PF-RC DNA levels from these mutants were clearly much lower than the WT (Fig 7A /lane 6, 7, 8 and 12/). Thus, the ratio of CCC DNA to PF-RC DNA in these mutants, as well as in S141A, E145D, and TT146/147/AA was significantly higher due to their stronger effects on PF-RC DNA vs. CCC DNA (Fig 7C).

**Fig 8:**
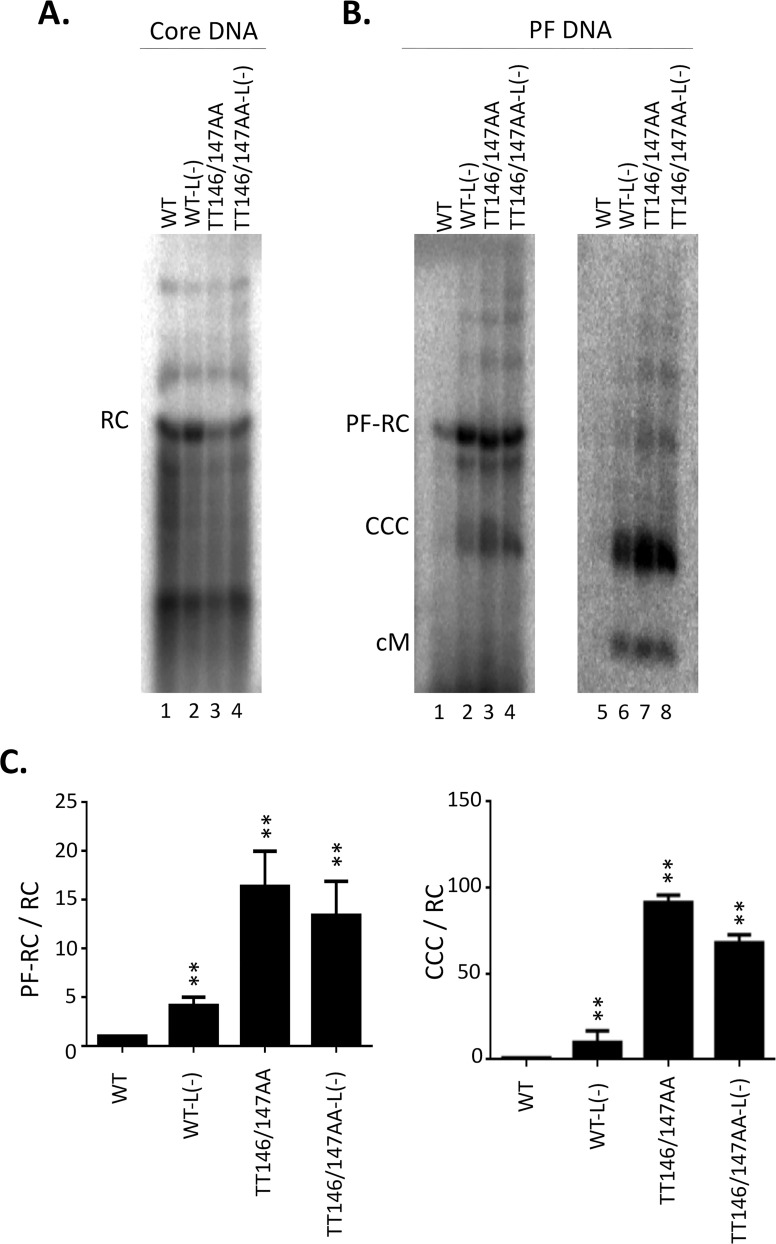
Analysis of CCC DNA from TT146/147AA in the presence and absence of the L protein. The full-length HBV replicon, with WT or TT146/147AA mutant HBc, or their L^-^ derivative was transfected into HepG2 cells. Transfected cells were harvested seven days post-transfection. **A.** HBV NC-associated DNA (core DNA) was released by SDS-proteinase K digestion from cytoplasmic lysates and detected by Southern blot analysis. **B.** PF DNA was extracted by Hirt extraction and digested with Dpn I (lane 1–4) or Dpn I plus exonuclease I and III (lane 5–8). RC, RC DNA; SS, SS DNA; PF-RC, PF-RC DNA; CCC, CCC DNA; cM, closed minus strand DNA. **C.** Quantitative results from multiple experiments. Left, PF-RC DNA normalized to core RC DNA; right, CCC DNA normalized to core RC DNA. All normalized values from the WT were set to 1.0. **, P<0.01.

Interestingly, an unusual smear of PF DNA, running between the PF-RC DNA and CCC DNA, was detected from those same four mutants (S141D, S141R, L143A, TT146/147/DD) that showed little to no RC DNA but yet comparable CCC DNA to WT (Fig 8A lane 6, 7, 8 and 12). Furthermore, the migration of this PF DNA smear was distinct in each mutant. The level of the smear seemed to be similar to that of CCC DNA but not PF-RC DNA for each mutant, suggesting that the formation of this DNA smear might be related to CCC DNA but not PF-RC DNA (see Discussion later).

As TT146/147AA did not appear to affect mature NCs stability (Fig 5B, lane 11), we were interested in determining if this mutant could increase CCC DNA levels (Fig 8A and 8B, lane 11) beyond its inhibitory effects on complete virion secretion (Fig 6A /lane 10/). Thus, we introduced the TT146/147AA linker mutation, without causing any amino acid change in the overlapping RT gene, into the full-length infectious HBV clone. In the full-length replicon context, TT146/147AA also showed increased CCC DNA levels (Fig 8B /lane 3 and 7/), similar to its effects in the *trans*-complementation assay above. To remove any effect of virion secretion on CCC DNA levels, we eliminated L protein expression in both the WT and TT146/147AA full-length clones. The RC DNA in mature NCs and protein-free DNA were increased in the L minus replicon compared with WT, as expected (Fig 8B /lane 2 and 6 vs. 1 and 5/). However, CCC DNA and PF-RC DNA levels in the replicon containing the TT146/147AA plus L minus mutation were still higher than those of the replicon containing the L minus mutation but WT HBc (Fig 8B /lane 4 and 8 vs. 2 and 6/ and 8C) but similar to those of TT146/147AA without the L mutation (Fig 8B /lane 3 and 7/). These results indicated that CCC DNA levels of TT146/147AA were not affected by elimination of L expression and thus, independent of secretion of complete virions. In cytoplasmic NCs, RC DNA levels in TT146/147AA and L minus replicon were lower than those in the L minus replicon containing WT HBc (Fig 8A /lane 4 vs. 2/), consistent with the notion that TT146/147AA might have enhanced RC DNA nuclear import in a manner independent of L (see Discussion also).

### Linker mutants blocked HBV infection in HepG2-NTCP cells

**Fig 9:**
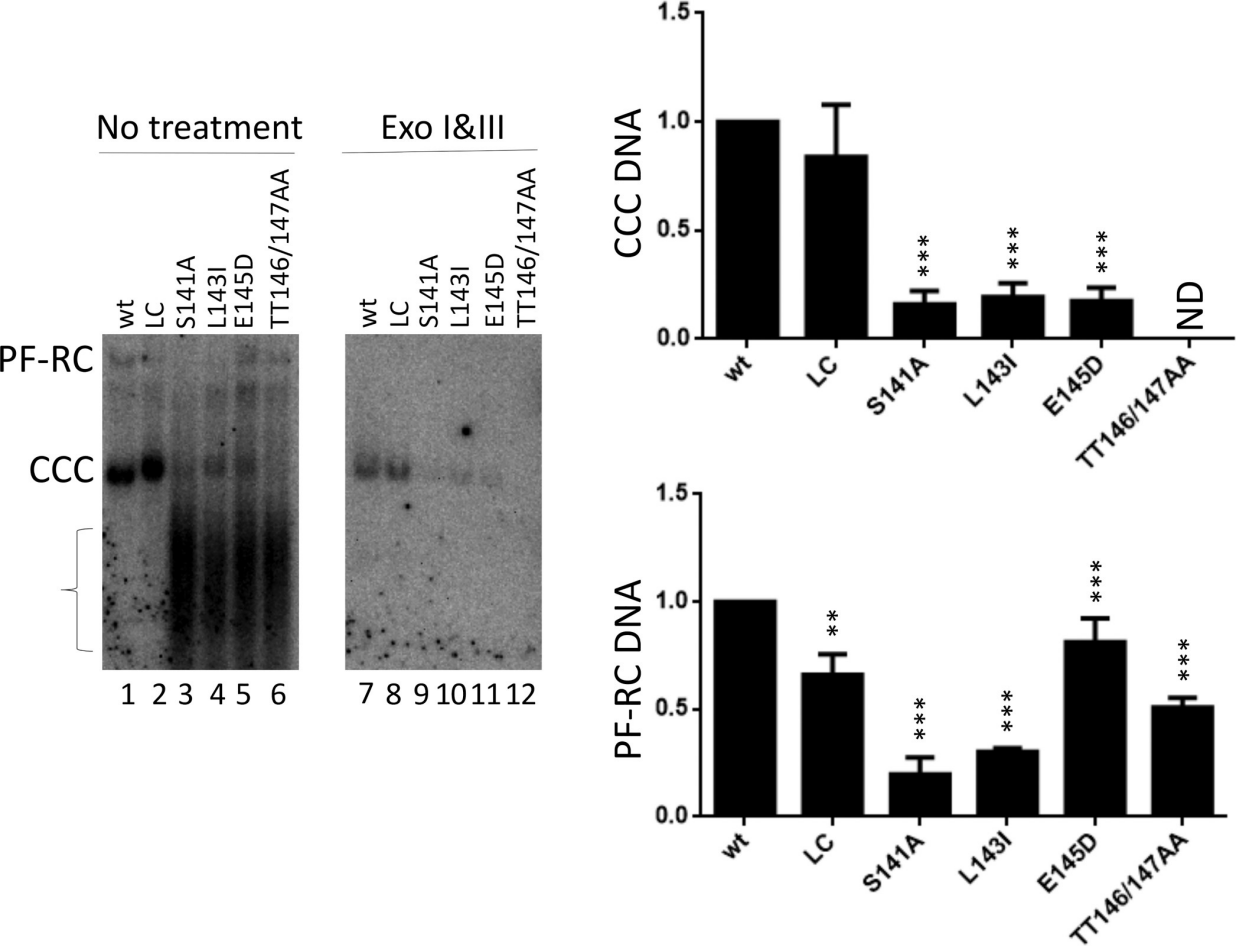
Effect of linker mutations on infection. HBV inocula were prepared from HepG2 cells co-transfected with the HBc-expression construct plus the HBc-defective replicon as shown in Fig 6, and used to infect HepG2-NTCP cells. PF DNA was extracted three days post-transfection and analyzed by Southern blot analysis, with (lane 1–6) or without (lane 7–12) pretreatment with exonuclease I and III (Exo I&III). Quantitative results are shown in the graphs to the right. The small m. w. DNA smear present in certain linker mutants (lane 3–6) is indicated by the bracket. PF-RC, PF-RC DNA; CCC, CCC DNA; ND, not detected. **, P<0.01;***, P<0.001.

To determine the infectivity of the linker mutants, viral particles harvested from HepG2 cells transfected with the WT or mutant HBc complemented with the HBc (-) HBV replicon (as shown in Fig 6), normalized to equal amounts of DNA-containing virions (at a MOI of 200), were used to infect HepG2-NTCP cells. PF DNA including CCC DNA was extracted from infected cells three days after infection and analyzed by Southern blot analysis, with or without pretreatment with Exo I and Exo III as we reported recently [61]. CCC DNA levels, taken as a measure of successful infection, produced by the LC mutant were similar to those of the WT (Fig 9 /lane 1, 2/). However, S141A, L143I, E145D all showed much reduced levels of CCC DNA (Fig 9 /lane 3–5 and 9-11/), and TT146/147AA showed no detectable CCC DNA (Fig 9 /lane 6,12/), indicating these four mutants severely impaired one or more steps in the infection process that leads to CCC DNA production. This was despite the fact that these same mutants all dramatically enhanced CCC DNA levels during intracellular amplification (Fig 7). These results thus clearly demonstrated that the linker mutations could differentially affect CCC DNA formation during infection vs. intracellular amplification. Interestingly, we detected a strong PF DNA smear (indicated by a bracket in Fig 9 /lane 3-6/) below CCC DNA from the four linker mutants that were defective in infection, which was absent from the WT or LC mutant that was infection-competent (Fig 9 /lane 1-2/), suggesting the production of the DNA smear may be somehow related to the block in infection (see Discussion also). The identity of the PF DNA smear remains to be better characterized but it was completely eliminated by the exonuclease digestion (Fig 9 /lane 9-12/), indicating it did not contain any covalently closed circular DNA—either SS or DS.

### Inhibition of PP2A activity decreased HBc dephosphorylation in a mammalian cell-free system

The above results suggested that the HBc linker may indeed function to recruit PP2A to modulate HBc phosphorylation and in turn, regulate HBV replication. To assess the possible role of PP2A, and potentially other cellular phosphatases, in HBc dephosphorylation, we took advantage of our recently developed RRL, cell-free, HBc expression and assembly system [29,62], in which the in vitro translated HBc is known to subject to dephosphorylation by a endogenous phosphatase(s). Furthermore, in the case of an HBc mutant, 3E, in which the three major CTD phosphorylation sites (S155, S162, and S170) are substituted with Glu, capsid assembly can be induced upon HBc dephosphorylation by the endogenous phosphatase. A major advantage of such a cell-free system is that various cellular phosphatases, which can be essential for numerous cellular processes, can be inhibited without concerns with potential cytotoxicity and pleiotropic effects in live cells treated with phosphatase inhibitors. An exogenous phosphatase such as CIAP can also be used readily as a dephosphorylation control [29]. HBc dephosphorylation can be monitored with mAbs selective for the dephosphorylated HBc CTD at the different sites (Fig 2A) [29]. In the case of the 3E HBc mutant, dephosphorylation can also be monitored conveniently by capsid assembly [29]. We selected two different phosphatase inhibitors with different selectivity (Table S1) [65,66]: fostriecin (PP2A/PP4 vs. PP1/PP5 selectivity ≥10^4^) and okadaic acid (PP2A/PP4 vs. PP1/PP5 selectivity <10^2^). Neither has any significant inhibitory activity against PP2B or PP7 and virtually no effect on PP2C or protein tyrosine phosphatases. Therefore, fostriecin can be employed in combination with okadaic acid to distinguishing the actions of PP2A/PP4 from PP1/PP5, which are the major classes of cellular Ser/Thr phosphatases.

**Fig 10:**
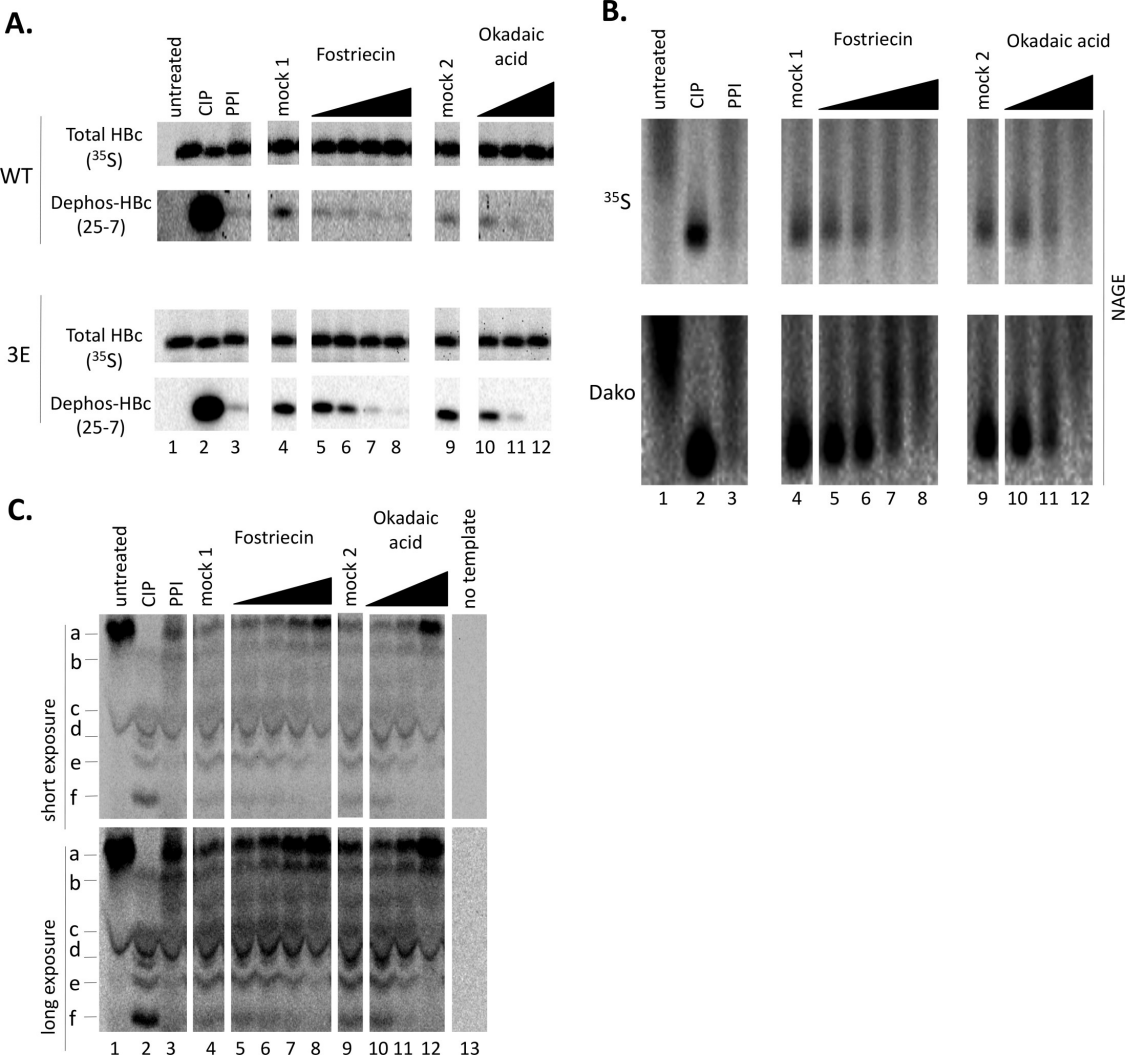
Effects of PP2A inhibitors on HBc dephosphorylation in vitro. The WT and 3E mutant HBc proteins were translated in RRL. The translated HBc, without any further treatment (untreated) or treated with NEBuffer 3 plus CIAP overnight at 37°C (CIP), with NEBuffer 3 plus a mixture of non-specific phosphatase inhibitors over night at 37°C (PPI), NEBuffer 3 alone overnight at 37°C (mock 1), with NEBuffer 3 plus 0.01% DMSO overnight at 37°C (CIAP) (mock 2), with NEBuffer 3 plus increasing concentrations of Fostriecin (10 nM, 20 nM, 40 nM, 80 nM) or Okadaic acid (1 nM, 10 nM, 100 nM), before further analysis. **A.** The ^35^S-labeled HBc proteins (WT and 3E) were resolved by SDS-PAGE and detected by autoradiography or western blot analysis using the HBc CTD dephosphorylation specific mAb 25–7. **B.** The HBc 3E reactions were resolved by NAGE. The ^35^S-labeled HBc proteins were detected by autoradiography (total HBc) or western blot analysis using the HBc specific polyclonal antibody (Dako). **C.** The HBc 3E reactions were resolved by Phos-tag SDS-PAGE, with the no-template translation reaction as a negative control, and ^35^S-labeled HBc proteins were detected by autoradiography. a & b: hyperphosphorylated 3E; c-e: hypophosphorylated 3E; f: non-phosphorylated 3E.

By comparing the signal intensity of HBc detected by mAb 25–7 vs. that of ^35^S-labeled (total) HBc detected by phosphorimaging, following SDS-PAGE and western blotting, it was clear that HBc was dephosphorylated at S178 (and potentially other sites as well) by the exogenous control CIAP (Fig 10A /top, lane 2/), and to a lesser degree, by the endogenous RRL phosphatase(s) (Fig 10A top /lane 4 and 9/), as we reported previously [29]. A cocktail of non-specific phosphatase inhibitors, employed here as an inhibitor control, could block HBc dephosphorylation by the RRL phosphatase (Fig 10A /top, lane 3 vs. 4 and 9/). Both specific phosphatase inhibitors, fostriecin and okadaic acid, could also block HBc dephosphorylation by the endogenous RRL phosphatase in a dose-responsive manner (Fig 10A /top, lanes 5–8 and 10-12/). In the case of the HBc 3E mutant, both specific inhibitors, as well as the non-specific phosphatase inhibitor cocktail, not only blocked its dephosphorylation (Fig 10A /bottom/) but also capsid assembly (Fig 10B), as expected from the ability of the endogenous RRL phosphatase to trigger 3E capsid assembly [29]. The effects of fostriecin and okadaic acid on 3E further indicated that the endogenous RRL phosphatase could dephosphorylate one or more of the four remaining S/T phosphorylation sites at its CTD (T160, S168, S176 and S178; Fig 2A), which was consistent with the detection of non-phosphorylated S178 by mAb 25–7. Based on the IC_50_ of these inhibitors (S1 Table) for the different phosphatases and the concentrations capable of inhibiting HBc dephosphorylation, we deduced that the endogenous cellular phosphatase(s) in the RRL responsible for dephosphorylating HBc was likely PP2A and/or PP4. Although specific inhibitors capable of distinguishing the actions of PP2A from PP4 are not yet available, PP2A was the most likely phosphatase candidate that mediated HBc CTD dephosphorylation in RRL, a cytoplasmic lysate, as PP4 is thought to function in the nucleus [67].

We also employed the Phos-tag SDS PAGE analysis to further determine the effects of the phosphatase inhibitors on the mutant 3E dephosphorylation in RRL in greater details. As we showed recently [28], 3E was phosphorylated in the remaining CTD sites in RRL as in human cells, migrating on the Phos-tag gel as predominantly a single hyperphosphorylated species (Fig 10C /lane 1, a/) and a couple of minor, less phosphorylated, species (Fig 10C /lane 1, b and c/). Treatment with CIAP led to the loss of hyperphosphorylated species (a) accompanies by the increase of the less phosphorylated species (b, c), and appearance of hypo-phosphorylated (d, e) and non-phosphorylated (f) species (Fig 10C /lane 2/). Mock treatment, without any exogenous phosphatase or inhibitors, i.e., dephosphorylation by the endogenous RRL phosphatase, caused similar effects as CIAP although dephosphorylation was not as extensive (Fig 10C /lane 4, 9/). A dose-responsive decrease of the hypo-phosphorylated HBc species (c-f) and a corresponding increase of the hyper-phosphorylated species (a-b) were caused by both fostriecin (Fig 10C /lane 5-8/) and okadaic acid (Fig 10C /lane 10-12/), similar to the effects of the non-specific phosphatase inhibitor cocktail control (Fig 10C /lane 3/). These results were thus in agreement with the results obtained using regular SDS-PAGE and western blot analysis with the CTD-dephosphorylation-specific mAb 25–7 (Fig 10A /bottom/). The effects of the inhibitors on multiple phosphor-HBc species, revealed by the Phos-tag gel, further suggested that PP2A likely dephosphorylated multiple CTD sites in RRL.

### Inhibition of PP2A could reduce HBc dephosphorylation and increase CCC DNA amplification in human cells

**Fig 11:**
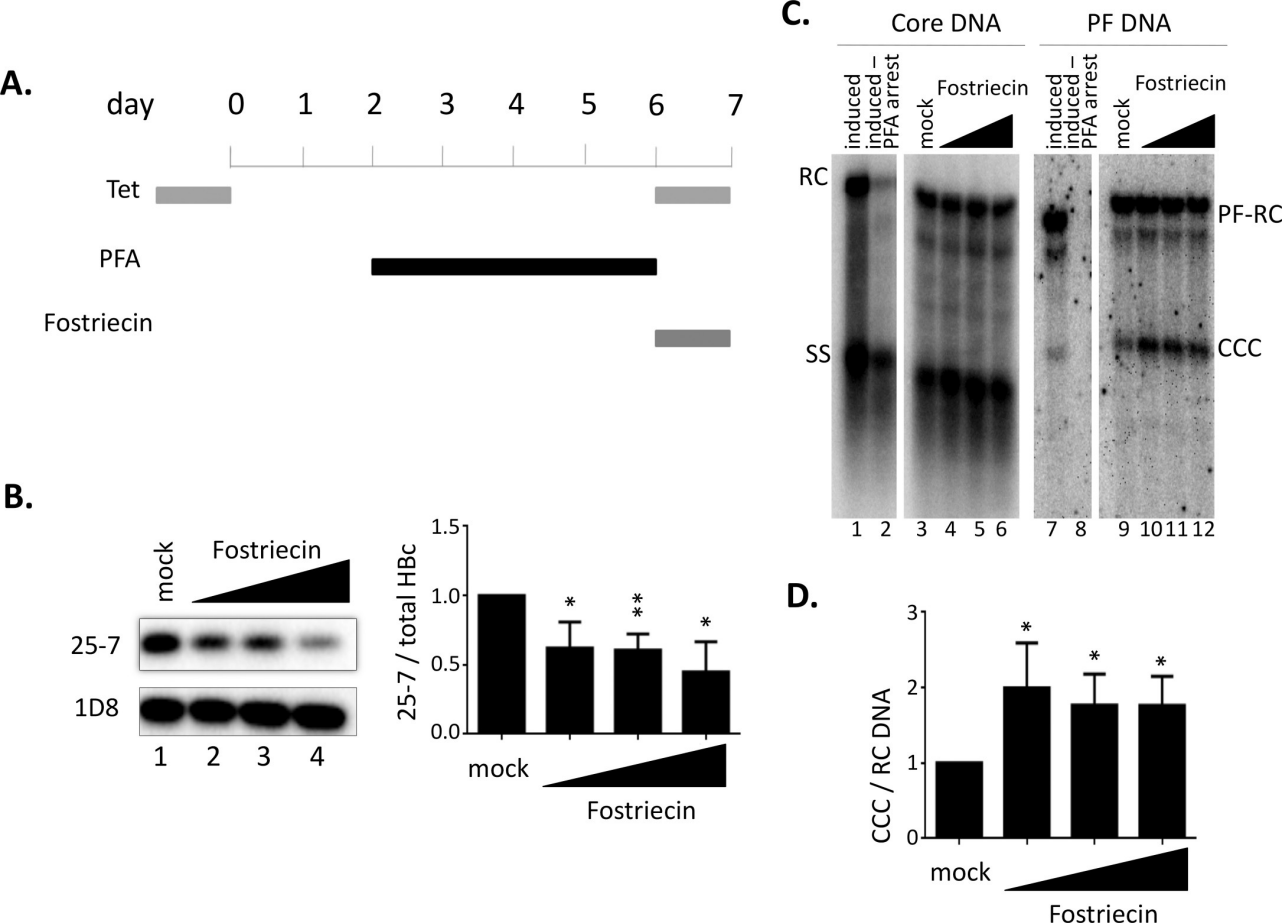
Effect of PP2A inhibition on HBc dephosphorylation and viral DNA replication in cell culture. A. Scheme for rapid and synchronized NC maturation and CCC DNA formation in cell culture. Following removal of tetracyclin (tet) from HepAD38 cells for two days to induce pgRNA expression packaging, PFA was added to arrest HBV DNA synthesis at the single-stranded (SS) DNA stage. Following 4 days of PFA treatment to accumulate SS DNA NCs, PFA was removed to allow rapid and synchronized synthesis of RC DNA from SS DNA and RC DNA conversion to CCC DNA during the ensuing one day before the cells were harvested. At the same time of PFA removal, tetracyclin was added back to turn off pgRNA synthesis and thus suppress further production of immature NCs. Fostriecin (20 μM, 40 μM, 60 μM) was added at the same time when PFA was removed. The cells were then harvested for analysis of HBc dephosphorylation (**B**) and viral DNA synthesis (**C**). **B.** HBc was resolved by SDS-PAGE and detected by western blot analysis, using the HBc CTD dephosphorylation (S178) specific mAb (25–7) or NTD mAb (1D8, for total HBc). The dephosphorylated HBc signal was normalized to total HBc signal. The normalized dephosphorylation signal of HBc treated with Fostriecin is shown in the graph relative to that of mock treatment, which was set to 1.00. **C.** HBV NC-associated DNA (core DNA, lane 1–6) and PF DNA (lane 7–12) were detected by Southern blot analysis. Lane 1 and 7 (induced): DNA from control cells cultured in the absence of tetracyclin or PFA for 7 days; lane 2 and 8 (induced-PFA arrest): DNA from cells harvested right at the end of PFA treatment before PFA release. RC, RC DNA; SS, SS DNA; PF-RC, PF-RC DNA; CCC, CCC DNA. **D.** Quantitative results of CCC DNA normalized to core RC DNA. The normalized CCC DNA signals from the cells treated with Fostriecin is shown relative to that of the mock treatment, which was set to 1.00. *, P<0.05; **, P<0.01.

Given the above results that PP2A inhibitors could decrease HBc dephosphorylation in the RRL system, we decided to test if PP2A also plays a role in HBc dephosphorylation in human hepatic cells. We have previously reported that NCs are dephosphorylated during maturation [31,33,44] but rephosphorylation of mature NCs may be needed to facilitate efficient uncoating to release RC DNA for CCC DNA formation [28]. Using the inducible HepAD38 cell HBV replication system that allows rapid and synchronous NC maturation and CCC DNA formation, based on reversible PFA arrest of HBV reverse transcription [28,59,63,64], we tested if inhibition of PP2A could affect NC maturation or CCC DNA formation (Fig 11A). Following the brief one-day treatment with the PP2A inhibitor fostriecin, HBc extracted from the treated cells was analyzed by SDS-PAGE and western blotting using the mAb 25–7 to detect the dephosphorylated (S178) HBc and mAb 1D8 targeting the HBc NTD (Fig 2A) to detect total HBc. We could indeed detect a dose-responsive decrease of HBc dephosphorylation upon treatment by fostriecin (Fig 11B). Furthermore, whereas the RC DNA level in the cytoplasm (i.e., level of NC maturation) was not affected significantly, CCC DNA was increased in a dose-responsive manner by fostriecin (Fig 11C and 11D). These results thus suggested that inhibition of NCs dephosphorylation, by PP2A, during maturation could promote uncoating of mature NCs to facilitate release of RC DNA and/or RC DNA nuclear import to enhance CCC DNA formation.

### Inhibition of PP2A could enhance PF-RC DNA during de novo infection

**Fig 12:**
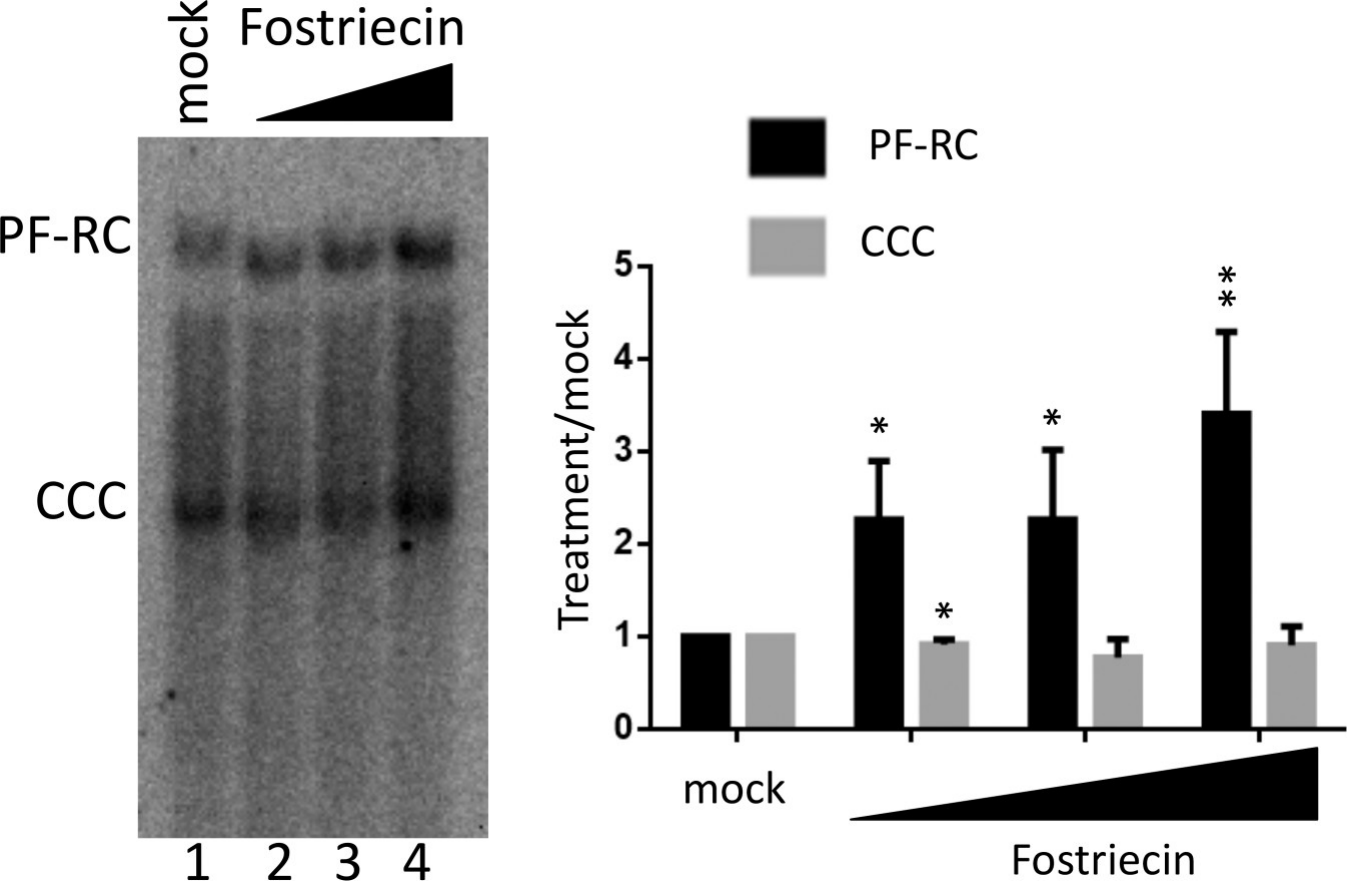
Effect of PP2A inhibition on HBV infection. HepG2-NTCP cells were infected with HBV for one day. Following removal of the inoculum, the cells were mock treated or treated with the PP2A inhibitor Fostriecin (20 μM, 40 μM, 80 μM). HBV PF DNA was extracted three days post-infection and detection by Southern blot analysis. PF-RC, PF-RC DNA; CCC, CCC DNA. In the graph, PF-RC DNA and CCC DNA signals from Fostriecin-treated cells were compared to mock treatment, which was set to 1.00. *, P<0.05; **, P<0.01.

To test if PP2A played a role in CCC DNA formation during infection, we determine the effects of PP2A inhibition on CCC DNA formation during HBV infection of HepG2-NTCP cells. As described in the Materials and Methods, HepG2-NTCP cells were pretreated with DMSO to arrest their growth so as to mimic the non-dividing hepatocytes in the human liver before infection. The cells were then infected with HBV and treated with the PP2A inhibitor fostriecin for three days. HBV PF DNA was extracted from the infected cells and analyzed by Southern blot analysis. We observed a dose-responsive increase in PF-RC DNA, though not CCC DNA, by fostriecin treatment (Fig 12), which was consistent with increased RC DNA deproteination due to enhanced uncoating, as a result of PP2A inhibition. Interestingly, CCC DNA was not significantly affected by fostriecin during infection (see more on this in Discussion below).

## Discussion

**Fig 13:**
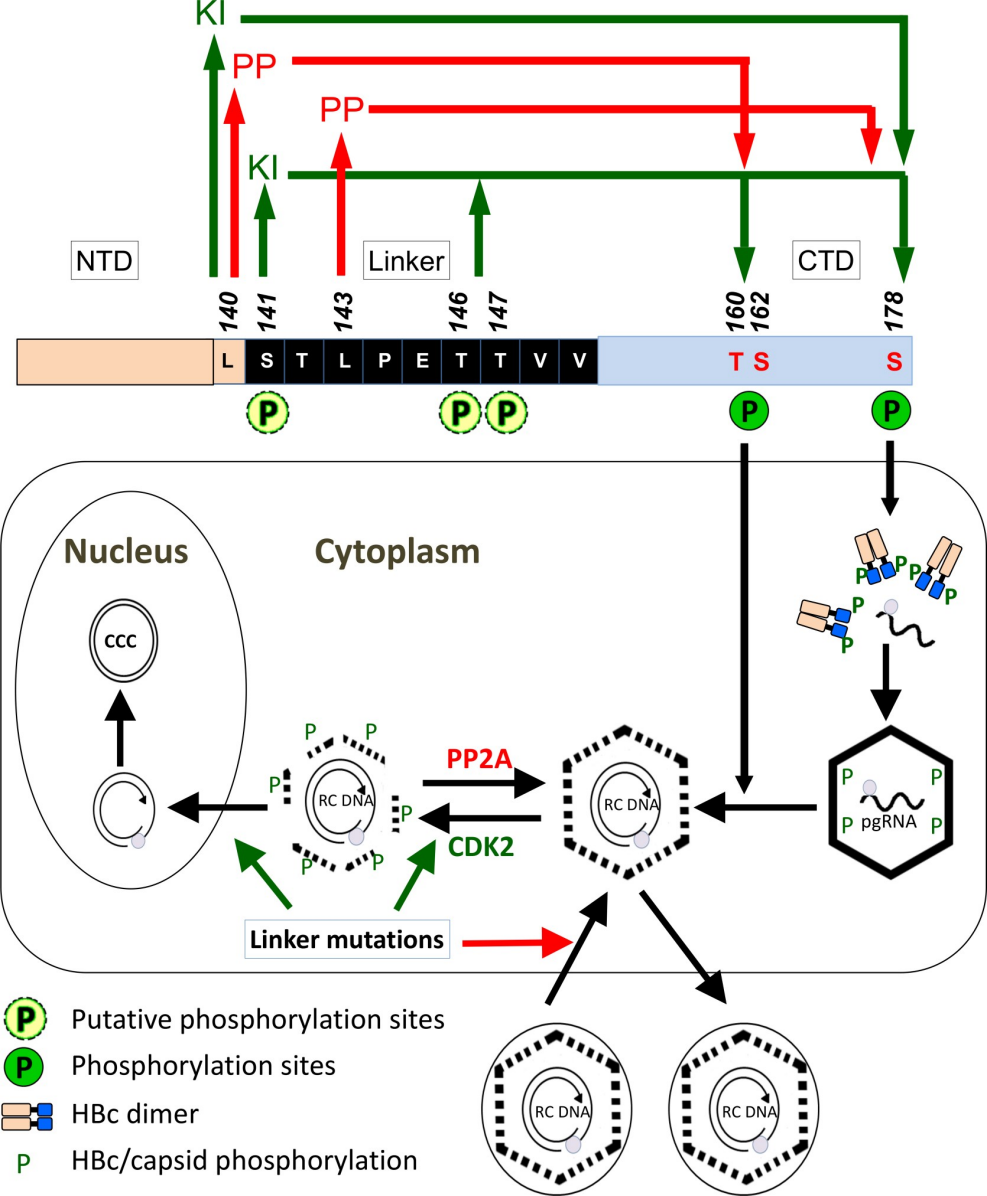
Model of interactions between HBc linker and host protein phosphatase (kinase) in regulating multiple steps of viral replication. The HBc domains (NTD, linker, CTD) are shown in the middle as horizontal boxes, with the linker sequence highlighted. Also highlighted are the last residue of NTD (L140, part of the PP2A-B56 consensus binding motif) and the three CTD sites where phosphorylation state was monitored in this study. The linker sequence is proposed to regulate dynamic CTD phosphorylation and dephosphorylation to control different stages of viral replication, among other mechanisms. The red and green arrows on the top denote the recruitment of cellular phosphatase (PP) and kinase (KI), respectively, by the indicated linker/NTD sites to modulate the indicated CTD phosphorylation sites. Shown at the bottom is a simplified scheme of HBV replication cycle, with the different stages affected by the different CTD phosphorylation sites and PP2A/CDK2 highlighted. The stimulatory and inhibitory effects of certain linker mutations on CCC DNA formation during intracellular amplification and infection, respectively, are denoted by the green and red arrows. See text for details.

The recognition of the consensus PP2A-B56 binding motif in the HBc linker (including the last residue of the NTD) and mutational analysis of the motif here provide support that the linker region plays a critical role in regulating HBc CTD phosphorylation state, in part by recruiting PP2A and possibly other cellular phosphatase(s) and even kinase(s) to modulate the dynamics of HBc CTD phosphorylation, in a site-specific manner, to control virtually every step of HBV replication. The differential effects of linker mutations on CCC DNA formation during intracellular amplification vs. infection highlights distinct regulation of these two pathways of CCC DNA formation and the role of HBc, specifically the linker region, in these processes. Effects of pharmacological inhibition of PP2A supports a role of PP2A in HBc dephosphorylation to regulate NC uncoating and CCC DNA formation (Fig 13).

Consistent with an important role of the highly conserved linker sequence (including the last residue of NTD), _140_LSTLPETTVV_149_, a near perfect match to the consensus PP2A-B56 binding motif (LxxIxE), in recruiting one or more phosphatases to dephosphorylate HBc, mutations within this sequence, designed to disrupt B56 binding, showed multiple effects on HBc phosphorylation state and viral replication (Fig 13). First, deletion of the entire linker sequence (141–149) profoundly decreased CTD dephosphorylation as determined by both mAbs, A701 for (non-phosphorylated) T160/S162 and 25–7 for (non-phosphorylated) S178, supporting an important role of the linker in facilitating CTD dephosphorylation at these CTD sites. Second, L140A and L143A, two critical residues in the motif for B56 binding, reduced HBc dephosphorylation, specifically at the A701 epitope (T160/S162), consistent with a role of the linker in recruiting PP2A-B56 to dephosphorylate these sites. Interestingly, whereas L143A also reduced S178 dephosphorylation as anticipated, L140A actually increased S178 dephosphorylation, suggesting that L140 may also normally play a role in recruiting a kinase to phosphorylate S178 (Fig 13). Furthermore, other mutations within the motif didn’t produce the expected effects based on linker recruitment of PP2A-B56. E145D, supposed to impair PP2A-B56 recruitment, showed no effect on CTD phosphorylation state, at least on the sites we could monitor with mAb A701 and 25–7. Also, S141D and TT146/147DD, designed to increase B56 binding to enhance CTD dephosphorylation, showed an opposite effect, decreasing dephosphorylation at both T160/S162 and S178. Admittedly, potential effects of the linker mutations on the other CTD phosphorylation sites not targeted by mAb A701 and 25–7 (i.e., other than T160, S162, and S178; Fig 2A) could not be assessed based on SDS-PAGE and western blot analysis. However, our Phos-tag gel analysis of S145D also didn’t reveal any significant change in HBc phosphorylation state, and S141D and TT146/147DD showed evidence of decreased, rather than the anticipated increased, dephosphorylation.

Thus, it appears unlikely that the linker directly recruits PP2A-B56 specifically, despite the presence of the consensus B56 binding motif. Consistent with this, we could not detect physical interaction between B56 and HBc/linker by immunoprecipitation, suggesting the linker may only interact with B56 transiently or weakly, if at all. Given that dynamic CTD phosphorylation and dephosphorylation are required for different stages of HBV replication, it is perhaps of no surprise that PP2A (or another phosphatase(s)), should be only recruited efficiently by HBc (linker) at certain stage of viral replication. It is also possible that B56 binding to the consensus motif is modulated by surrounding/neighboring sequences. In addition, as little is currently known about the recruitment of other PP2A regulatory (B) subunits, it remains possible that the “B56 binding motif” in the HBc linker may actually bind to another PP2A B subunit. Furthermore, the fact that different linker mutations affected the different CTD phosphorylation sites differently is consistent with the notion that multiple phosphatases (and possibly kinases, see below) may be recruited by the linker to modulate HBc phosphorylation state, which could confound the interpretation of the linker mutational effects on HBc phosphorylation state.

Our observation that the phosphomimetic linker mutants, T141D and TT146/147DD, may promote CTD phosphorylation suggest that the linker, when phosphorylated, may also serve to recruit a kinase(s) to phosphorylate CTD (Fig 13), or alternatively, linker phosphorylation may enhance CTD phosphorylation by some other means, e.g., by inducing structural changes in HBc. Interestingly, we have recently found that HBc NTD phosphorylation may also enhance CTD phosphorylation [28]. In support of this cross-regulation mechanism, our previous results [28,29] and here (S1 Fig) clearly showed that blocking CTD phosphorylation at the three major sites (all SP motifs, Fig 2A), as in the 3A mutant, dramatically enhanced phosphorylation in one or both of the SQ sites (S168, S176) while decreasing phosphorylation at the other minor CTD sites (S178, and possibly also T160). As we have recently shown that little HBc phosphorylation occurs outside the CTD in human cells under steady state [28], the putative linker phosphorylation would occur only transiently, e.g., during NC uncoating.

Dynamic HBc CTD phosphorylation and dephosphorylation are generally thought to play a critical role in pgRNA packaging and reverse transcription (Fig 13). This has been largely based on mutagenesis studies targeting the sites of phosphorylation to mimic non-phosphorylation (Ala) or constitutive phosphorylation (Asp or Glu) [30,31,38,39,68–71], which does not allow for modeling of dynamic change in phosphorylation state and furthermore, can be subject to alternative interpretations such as the effects of the identity of the targeted residue *per se* vs. its state of phosphorylation. Biochemical studies to establish correlation of phosphorylation or non-phosphorylation at particular sites with a functional state, when feasible, can complement the genetic studies to decipher the role of a particular phosphorylation site and furthermore allow monitoring of dynamic state [21,33,41,42,44]. Here, we have demonstrated that the phosphorylation state of the HBc CTD, as modulated by mutagenesis of the linker, without any changes to the CTD phosphorylation sites themselves, can be correlated to pgRNA packaging or RC DNA synthesis (Fig 1B), thus providing independent support for the roles of CTD (de)phosphorylation in these processes.

Strikingly, the subtle change at the last residue of the NTD, L140A profoundly blocked pgRNA packaging. Similarly, the linker deletion mutant also strongly inhibited pgRNA packaging (this study and [40]). However, the CTD phosphorylation state between these two mutants were very different. The linker deletion mutant showed strongly decreased CTD dephosphorylation at both T160/S162 and S178, whereas L140A caused a dramatic increase in dephosphorylation at S178 while decreasing CTD dephosphorylation at T160/S162. The other linker mutants with decreased dephosphorylation (S141D, S141R, L143A, TT146/147/DD) at T160/S162 did not affect pgRNA packaging significantly. These results suggest that the role of CTD dephosphorylation in pgRNA packaging [41], potentially mediated by the protein phosphatase 1 (PP1) as reported very recently [42], is site-dependent. T160/S162 dephosphorylation may not be necessary for pgRNA packaging, and S178 may in fact need to be phosphorylated to facilitate pgRNA packaging. This is consistent with previous reports showing that S162A, but not S162D, can strongly inhibit (by 80%) pgRNA packaging [30,38,43,71]. Also, our Phos-tag gel analysis showed that L140A phosphorylation was highly heterogeneous, in contrast to the fairly uniform hyperphosphorylation of the WT HBc. whether this heterogeneity contributes to the effects on pgRNA packaging is not yet clear. Additionally, possible structural effects of the linker mutants on HBc and the assembling capsid, which in turn affects pgRNA packaging, warrant further studies.

Similar to pgRNA packaging, SS DNA synthesis was not significantly affected by most of the mutants here. The only mutant showed little to no SS DNA was L140A, which was severely defective in pgRNA and was therefore expected to make little SS DNA. These results are consistent with our recent report that the linker facilitates pgRNA packaging and SS DNA synthesis in a sequence-independent manner [40]. In sharp contrast, several linker mutants showed little to no RC DNA (Fig 1B), consistent with our recent report that the linker facilitates RC DNA synthesis in a sequence-dependent fashion [40]. In particular, T160/S162 dephosphorylation could be correlated strongly with RC DNA levels in cytoplasm NCs. Three mutants (S141D, L143A and TT146/147DD) with no detectable cytoplasmic RC DNA signal also showed severely reduced dephosphorylation of T160/S162. The five mutants (S141A, L143I, E145D, TT146/147AA and LC) that didn’t affect the T160/S162 state of phosphorylation also showed little to no effect on RC DNA. These results thus support our previously proposed model, based on mutagenesis and biochemical studies of the DHBV core [31,33] and biochemical studies of HBc [44], that NC dephosphorylation following SS DNA synthesis is critical for the production of RC DNA and the stability of mature NCs. An important role of T160 and S162 dephosphorylation in RC DNA synthesis was also suggested by previous mutagenesis studies. Thus, HBc S162D appeared to preferentially impair RC DNA synthesis over that of SS DNA [70]. Interestingly, HBc T160A (T162A in strain adw) is the only mutation on the minor phosphorylation sites that almost completely blocks RC DNA formation [69], suggesting that premature T160 dephosphorylation may inhibit RC DNA synthesis. Similarly, S141R, the linker mutant here that enhanced dephosphorylation at T160/S162, also reduced RC DNA. These results imply that dephosphorylation of S162 is synchronized with plus strand DNA elongation to ensure mature RC DNA formation and/or accumulation, consistent with our previous biochemical characterization of NC phosphorylation state in both DHBV and HBV [33,44].

As we reported previously with certain HBc NTD mutants [27], the loss of RC DNA in some of the linker mutants might be due to the loss of stability/integrity of RC DNA-containing NCs so that their RC DNA content was degraded, in addition to, or other than, a defect in RC DNA synthesis. Indeed, mature NCs of S141A, S141R and L143I failed to protect their low levels of RC DNA from DNase digestion, indicative of a loss of NC integrity. Furthermore, the immature DS DNA species migrating below the mature RC DNA, presumably representing incomplete RC DNA with relatively short plus strands, was also digested by DNase in these mutants and the exact species digested appeared to vary depending on the linker mutation, e.g., L143I retained the DNA species migrating just below RC DNA that was lost in S141A and S141R after DNase digestion (Fig 5B), suggesting a finely tuned modulation of NC stability by the linker during maturation. The effect of the linker mutations on mature NCs stability, may be, at least in part, mediated through their influences on CTD phosphorylation state [31] as well as possible structural/conformational effects.

Our previous studies have shown deletions or substitutions of linker could strongly influence empty virion secretion [40], but effects of the linker on secretion of complete virions was unclear since most of the previous linker mutants failed to make RC DNA, a prerequisite for secretion of complete virions, and the one mutant able to make RC DNA was also competent to secret complete virions [40]. In this study, E146D and TT146/147AA mutants, which showed relatively high levels of RC DNA in cytoplasmic NCs, secreted very little to no DNA virions, indicating that the linker sequence can also affect secretion of DNA virions by affecting the interaction between the mature NC and the envelope, directly or indirectly. There was little correlation between levels of complete virions and the CTD phosphorylation state (Fig 2B), suggesting that regulation of complete virion secretion is independent of CTD phosphorylation state per se, consistent with our recent report that CTD dephosphorylation, though associated with NC maturation, is not essential for the subsequent NC-envelope interaction to secrete DNA-containing or empty virions [44]. Possibly, the linker exerts its effects on the secretion of complete virions through affecting the structure of the mature NC and hence indirectly affecting NC interaction with the envelope proteins. For empty virions, all linker mutations here as well as those we reported previously [40] severely impaired their secretion, further highlighting the critical role of the linker in supporting secretion of empty virions, possibly through direct linker interactions with the envelope proteins.

All of the linker mutants tested here, except for the linker deletion mutant Δ141–149 and L140A, both of which were severely defective in pgRNA packaging and thus showed little DNA synthesis, remained competent for CCC DNA formation. In fact, even those with no detectable RC DNA in cytoplasmic NCs, showed levels of CCC DNA similar to, or increased over, the WT. The mature NCs of S141A and L143I were sensitive to DNase, suggesting enhanced uncoating might be responsible for their increased CCC DNA and PF-RC DNA levels, similar to the NTD mutants L60A and I126A [27]. E145D and TT146/147AA, which did not significantly affect the integrity of mature NCs, also strongly enhanced CCC DNA and PF-RC DNA levels; Their decreased secretion of DNA virions could be partially responsible [11–13]. However, both the PF-RC DNA and CCC DNA levels from TT146/147AA were much higher than the L-defective mutant (with WT HBc, unable to secrete DNA virions), and eliminating L expression from TT146/147AA didn’t enhance CCC DNA or PF-RC DNA levels further. Thus, TT146/147AA may increase CCC DNA and PF-RC DNA mainly by enhancing nuclear import of RC DNA, as we suggested before for the HBc NTD mutant K96A [27]. Whereas the nuclear localization signals (NLSs) are localized on the HBc CTD arginine rich domain [37,72], it’s possible that the linker could regulate the exposure or function of the CTD NLSs through affecting the CTD phosphorylation state [21,37] or the structure of mature NCs [73].

Perhaps the most intriguing mutants identified here were S141D, S141R, L143A and TT146/147DD, which showed little to no RC DNA in cytoplasmic NCs, could nevertheless still produce CCC DNA at similar levels to or even higher than WT. Also, the PF-RC DNA levels of these four mutants were much lower than WT, in contrast to all other CCC DNA-enhancing HBc mutants here and in our previous studies [27,28] that always increased PF-RC DNA. Furthermore, these four mutants all produced a novel PF-DNA smear migrating between PF-RC and CCC DNA. These results suggest very rapid conversion of RC DNA to PF-RC DNA in these linker mutants, possibly due to rapid uncoating and nuclear import of these mutant NCs, allowing little to no cytoplasmic mature NC accumulation. Some of the released RC DNA from such hyper-destabilized mature NCs may also have been rapidly degraded, generating the PF-DNA smear, before it could be converted to PF-RC DNA and CCC DNA. The immature PF DNA smear could also result from premature uncoating of NCs prior to the synthesis of mature RC DNA, with their immature DS DNA becoming deproteinated. Whether these prematurely deproteinated DS DNA can be converted to CCC DNA or not is an interesting question that warrants further investigation. The putative rapid and premature uncoating in these mutants could also explain their failure to interact with the envelope proteins for virion secretion, and their decreased levels of PF-RC DNA despite WT-like or higher levels of CCC DNA. It is also notable that the maturity of the PF DS DNA, presumably representing the exact timing of premature uncoating and deproteination or degree/nature of RC DNA degradation, also varied with each of these mutants, further highlighting the critical role of the specific linker sequence in controlling the uncoating/RC DNA deproteination process.

In contrast to their enhanced CCC DNA formation via intracellular amplification, the linker mutants S141A, L143I, E145D, and TT146/147AA blocked CCC DNA formation during infection. These are the first HBV mutants reported so far to have opposite effects on CCC DNA formation during infection vs. intracellular amplification (Fig 13). Interestingly, certain small molecules targeting the capsid also showed similar effects, enhancing CCC DNA formation during intracellular amplification but block CCC DNA formation during infection [74]. These results thus clearly indicate that the capsid can regulate CCC DNA formation differentially during infection vs. intracellular amplification, the mechanism of which requires further studies. These four linker mutants, defective in CCC DNA formation during infection, also produced a strong, fast-migrating PF DNA smear, which was absent in WT or the LC mutant that showed no defect in infection. This result suggests possible degradation of input RC DNA from these four mutants during infection, which may be related to the hyper-destabilization of mature NCs in S141A and L143I. However, in the case of E145D and TT146/147AA in which mature NCs did not show apparent hyper-destabilization, a different mechanism(s), e.g., nuclear import of RC DNA, may be implicated (Fig 13).

We could effectively monitor only dephosphorylation at two or three CTD phosphorylation sites (i.e., mAb A701 for T160/S162 and mAb 25–7 for S178) by SDS-PAGE and western blot analysis, due to the lack of antibodies selective to the unphosphorylated state at the other phosphorylation sites. It is certainly possible, even likely, that dephosphorylation at these other sites were also affected by the different mutations tested here, which could contribute to the phenotypes observed. Also, although the Phos-tag gel analysis was able to reveal much more complex effects on HBc phosphorylation state by the mutants beyond those revealed by regular SDS-PAGE, the functional consequences of these complex effects on HBc phosphorylation state are not entirely clear or straightforward to interpret at present. Future studies employing additional site-specific and phosphor-selective antibodies will be required to define each of the multiple bands resolved by the Phos-tag gels in terms of their state of phosphorylation at each site of the HBc CTD (and potentially the NTD and linker as well) and to correlate their abundance to HBc functions at various stages of HBV replication. On the other hand, our results here do show that the Phos-tag gel analysis can provide important additional information to relate HBc phosphorylation state to HBc functions, beyond what can be obtained using regular SDS-PAGE analysis. For example, the lower degree of phosphorylation in Δ141–149 than L143A, revealed clearly only by the Phos-tag gels but not by regular SDS-PAGE, may be related to their respective effects on pgRNA packaging (by Δ141–149) vs. RC DNA levels (by L143A). Similarly, the lower degree of phosphorylation in L143I than S141D, again revealed by the Phos-tag gel but not by regular SDS-PAGE, may be related to their differential effects on RC DNA. Future technological advances will also be required to facilitate efficient separation of empty HBV capsids as well as NCs with different maturity so that NC phosphorylation state can be assessed as a function of maturation. Moreover, the various mutants here could exert subtle structural effects that in turn could contribute to the observed functional effects, which will require detailed structural studies across the different stages of HBV NC maturation in the future.

The critical roles of the HBc linker in viral replication is reflected in the high degree of sequence conservation in this region as shown in a previous report [75]. We have updated this analysis by using a total of 11,301 HBc sequences belonging to different HBV genotypes available in the HBVdb database and the Clustal Omega sequence alignment program. Our analysis showed that the N-terminal part of the HBc linker (141–145) and the last residue of the NTD (L140), which harbor the critical residues within the PP2A binding motif, are highly conserved (S2 Table), even more so than the C-terminal part of the linker. Interestingly, the L143I substitution studied here was found to occur naturally (albeit only at 0.079% frequency) around the world (such as China AMQ47839.1, India CDW17288.1 and West-African CAM58697.1). Similarly, the A147T substitution, included in our double mutant TT146/147AA, was detected in a number of clinical isolates (such as ACT90732.1, AIW67706.1, and AKJ76200.1). Any effects of these substitutions on the clinical outcomes of infection warrant further studies in light of our results here. Some linker mutations, e.g., those that showed severe defect in virion secretion (S141D, S141R, L143A), may not be detectable even if occurring naturally, since only blood samples are usually used in sequence surveys.

Although the exact PP2A isoform(s) recruited to dephosphorylate the HBc CTD remains to be clarified, our results suggest that PP2A is one of the major cellular phosphatases responsible for CTD dephosphorylation. In addition to the effects of HBc linker mutations within the B56 consensus binding motif, specific pharmacological inhibitors of PP2A could block HBc dephosphorylation in vitro and in vivo, at least at S178 and likely other sites. Furthermore, inhibition of PP2A-mediated HBc dephosphorylation could be correlated with increased CCC DNA levels, suggesting PP2A functions to restrain CCC DNA formation. Our recent study indicates that the HBc CTD is dephosphorylated in mature NCs [44], as in DHBV [31,33] but subsequently, rephosphorylation of mature NCs, likely mediated by the packaged CDK2 (the endogenous kinase), may facilitate disassembly (uncoating) of mature NCs to release RC DNA for CCC DNA formation [28]. Therefore, PP2A, by dephosphorylating mature NCs to enhance their stability, may play a role, opposite to CDK2, to restrain NC uncoating and CCC DNA formation (Fig 13). In addition, PP2A dephosphorylation of the HBc CTD may also modulate nuclear import of RC DNA to influence CCC DNA formation. In addition, pharmacological inhibition of PP2A implicated a role for PP2A-mediated HBc dephosphorylation in facilitating capsid assembly the RRL cell-free system. The role of PP2A in other steps of HBV replication, as suggested by the genetic analysis of the B56 binding motif in the linker, requires further verification.

It is interesting that decreased CTD dephosphorylation due to PP2A inhibition, leading to enhanced CCC DNA level during intracellular amplification, did not significantly reduce the RC DNA levels in mature NCs, generated from the SS DNA and incomplete DS DNA during the short synchronized DNA synthesis period, suggesting that PP2A-mediated CTD dephosphorylation may not affect significantly plus strand DNA elongation and instead mostly affected the subsequent disassembly (uncoating) of the mature NC [28,31,44]. As only a small fraction (5% or less) of RC DNA from in mature NCs is converted to CCC DNA [11], a modest (2-fold) increase in CCC DNA, as a result of PP2A inhibition, may not be reflected by a significant decrease in RC DNA in mature NCs. The increase in PF-RC DNA during infection caused by PP2A inhibition is also consistent with the stabilizing effect of PP2A-mediated NC dephosphorylation. On the other hand, CCC DNA was not significantly affected by PP2A inhibition during infection, suggesting that an additional rate-limiting step(s) in CCC DNA formation, beyond NC uncoating, during infection but not during intracellular amplification, that was not overcome by PP2A inhibition (Fig 13). This is also consistent with the differential effects on CCC DNA formation during intracellular amplification vs. infection of the linker mutations here (Fig 13) and core inhibitors reported recently [74].

It has been proposed that HBc may also play a role in the host cell nucleus, including regulation of CCC DNA transcription, in particular, the HBV core promoter [76,77] and involvement in CCC DNA degradation [78], but these remain controversial [79,80]. In transient transfection assays such as those we used here, HBV transcription is almost exclusively driven from the transfected plasmid DNA, with little contribution from CCC DNA. In addition, in this study, the HBV pgRNA was expressed not from the HBV core promoter but from the heterologous human cytomegalovirus promoter. Furthermore, we observed no significant differences in expression among the WT and mutant HBc proteins whether CCC DNA levels were deceased or increased as compared with the WT. We have not examined the effects of our HBc linker mutants on the reported nuclear functions of HBc, which may be warranted in future studies.

In conclusion, our results have demonstrated the essential and multiple roles of the HBc linker in HBV replication, extending our recent discovery on linker functions [40]. Furthermore, the identification of a consensus PP2A-B56 binding motif spanning the end of the NTD and most of the linker, and our mutagenesis analysis of this motif support the notion that the linker exerts its multiple functions, in part, through the recruitment of a cellular phosphatase(s) to modulate the phosphorylation state of the CTD, and possibly that of the NTD [28] and linker itself. The effects of pharmacological inhibition of PP2A also support a role of PP2A in dephosphorylating the HBc CTD, which in turn, regulates NC stability and uncoating. However, our mutagenesis results were not fully consistent with a role of specifically the PP2A-B56 holoenzyme. Instead, our results suggest that another related PP2A holoenzyme containing a targeting (regulatory) subunit with similar but not identical binding motif might be recruited by the linker. Furthermore, these results suggest that additional phosphatases, e.g., the recently reported PP1 [42], and possibly kinases and other host factors, may also be recruited to modulate the CTD phosphorylation state in a site-specific manner, which, together with potential structural effects, regulate multiple steps of HBV replication.

## Supporting information

S1 Fig Probable recognition of the non-phosphorylated S178 in HBc CTD by mAb 25–7. **A.** Schematic of HBc domain structure and mAb (1D8 and 25–7) epitopes. The CTD sequence of the genotype D strain used here is shown at the bottom, with the three major and four minor sites of phosphorylation highlighted. **B.** HepG2 and KEK293 cells were transfected with the expression plasmid for WT HBc or an HBc mutant 3A, in which the three major phosphorylation sites (S155, S162 and S170) are substituted by Ala. Cytoplasmic lysates from transfected cells were resolved by SDS-PAGE and the HBc proteins were detected by western blot analysis, using the NTD-specific mAb 1D8, and the CTD-specific mAb 25–7, and the pS-Q mAb that recognizes phospho-Ser followed by Gln (i.e., S168 and S176 in CTD). **C** & **D.** Cytoplasmic lysates of HepG2 cells transfected with the expression plasmid for WT (**C**) or the indicated mutant HBc protein (**C** & **D**) were resolved by NAGE and the HBc proteins were detected by western blot analysis, using the NTD-specific mAb T2221 (**C**), the polyclonal HBc antibody (Dako) (**D**), or the mAb specific for the pSQ motif (**C** & **D**). HBc protein purified from E. coli was loaded as non-phosphorylated control (**C**). The 3A, 3E, 7A, or 7E mutant HBc has the three major phosphorylation sites (S155, S162 and S170) substituted by Ala (3A) or Glu (3E), or all seven CTD phosphorylation sites substituted by Ala (7A) or Glu (7E).
(TIF)

S1 Table Inhibitors of Ser/Thr Protein Phosphatase (IC_50_)* *The IC50 values provided are nM and represent the concentration of inhibitor needed to inhibit 50% of the activity of the respective enzyme. This table is adapted from Honkanen and Golden [66]. ND: not determined.
(DOCX)

S2 Table Conservation of HBc Linker Residues* *A total of 11,301 HBc sequences belonging to different HBV genotypes were downloaded from the HBVdb database and analyzed using the Clustal Omega sequence alignment program. The degree of conservation at the last position of the HBc NTD (140) and within the linker peptide (141–149) is shown. The frequency of I143 and A147, which were included in the current mutagenesis study, is shown in parentheses.
(DOCX)
