# Supplementary figures and images for: Multiple roles of PP2A binding motif in hepatitis B virus core linker and PP2A in regulating core phosphorylation state and viral replication
Source: PLoS Pathog. 2021 Jan 25;17(1):e1009230. doi: 10.1371/journal.ppat.1009230 (PMC7861550; doi:10.1371/journal.ppat.1009230)

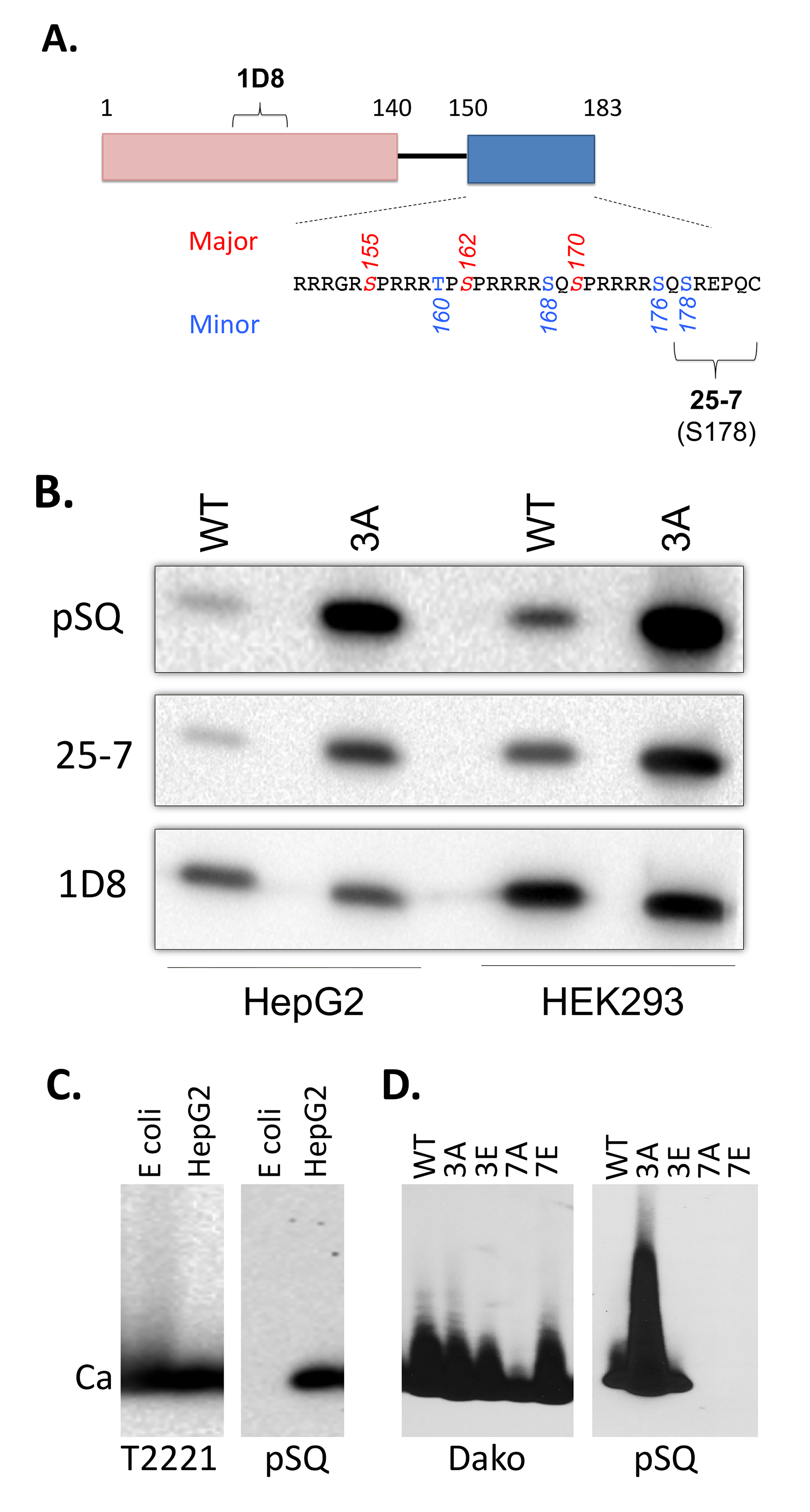

Supplement: S1 Fig — A. Schematic of HBc domain structure and mAb (1D8 and 25–7) epitopes. The CTD sequence of the genotype D strain used here is shown at the bottom, with the three major and four minor sites of phosphorylation highlighted. B. HepG2 and KEK293 cells were transfected with the expression plasmid for WT HBc or an HBc mutant 3A, in which the three major phosphorylation sites (S155, S162 and S170) are substituted by Ala. Cytoplasmic lysates from transfected cells were resolved by SDS-PAGE and the HBc proteins were detected by western blot analysis, using the NTD-specific mAb 1D8, and the CTD-specific mAb 25–7, and the pS-Q mAb that recognizes phospho-Ser followed by Gln (i.e., S168 and S176 in CTD). C & D. Cytoplasmic lysates of HepG2 cells transfected with the expression plasmid for WT (C) or the indicated mutant HBc protein (C & D) were resolved by NAGE and the HBc proteins were detected by western blot analysis, using the NTD-specific mAb T2221 (C), the polyclonal HBc antibody (Dako) (D), or the mAb specific for the pSQ motif (C & D). HBc protein purified from E. coli was loaded as non-phosphorylated control (C). The 3A, 3E, 7A, or 7E mutant HBc has the three major phosphorylation sites (S155, S162 and S170) substituted by Ala (3A) or Glu (3E), or all seven CTD phosphorylation sites substituted by Ala (7A) or Glu (7E). (TIF) [file ppat.1009230.s001.tif]
