# Supplementary material for: Multiple roles of PP2A binding motif in hepatitis B virus core linker and PP2A in regulating core phosphorylation state and viral replication
Source: PLoS Pathog. 2021 Jan 25;17(1):e1009230. doi: 10.1371/journal.ppat.1009230 (PMC7861550; doi:10.1371/journal.ppat.1009230)
Supplement: S1 Table — This table is adapted from Honkanen and Golden [66]. ND: not determined. (DOCX) [file ppat.1009230.s002.docx]

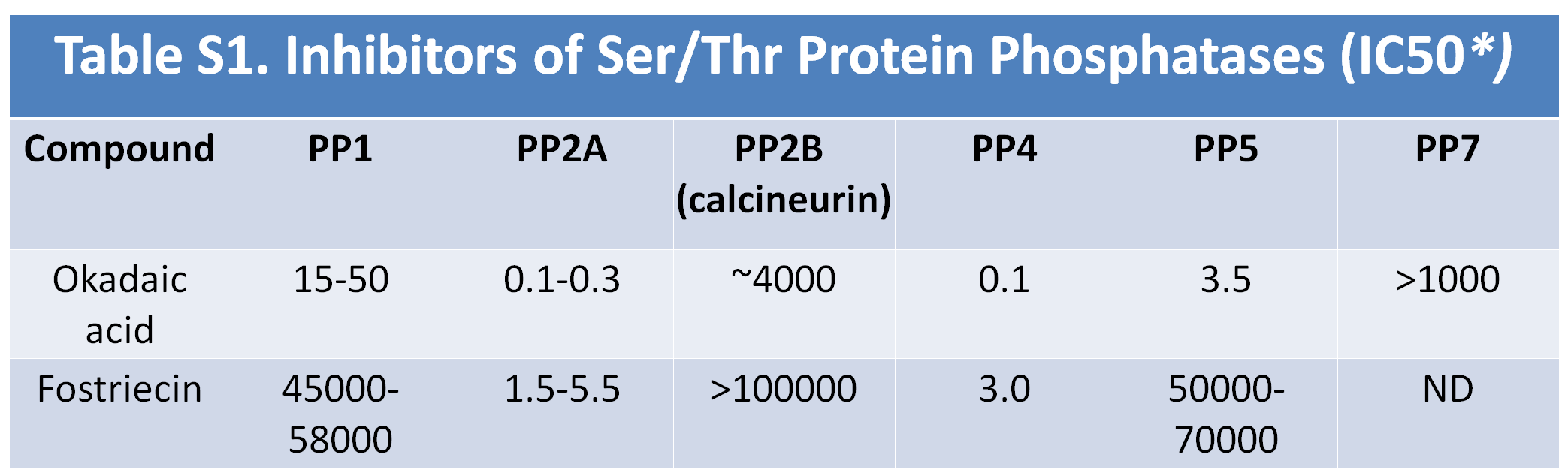


*The IC50 values provided are nM and represent the concentration of inhibitor needed to inhibit 50% of the activity of the respective enzyme. This table is adapted from Honkanen and Golden [1].

ND: not determined

**Reference**

1. Honkanen R, Golden T (2002) Regulators of serine/threonine protein phosphatases at the dawn of a clinical era? Current medicinal chemistry 9: 2055-2075.
