# Supplementary material for: Multiple roles of PP2A binding motif in hepatitis B virus core linker and PP2A in regulating core phosphorylation state and viral replication
Source: PLoS Pathog. 2021 Jan 25;17(1):e1009230. doi: 10.1371/journal.ppat.1009230 (PMC7861550; doi:10.1371/journal.ppat.1009230)
Supplement: S2 Table — The degree of conservation at the last position of the HBc NTD (140) and within the linker peptide (141–149) is shown. The frequency of I143 and A147, which were included in the current mutagenesis study, is shown in parentheses. (DOCX) [file ppat.1009230.s003.docx]

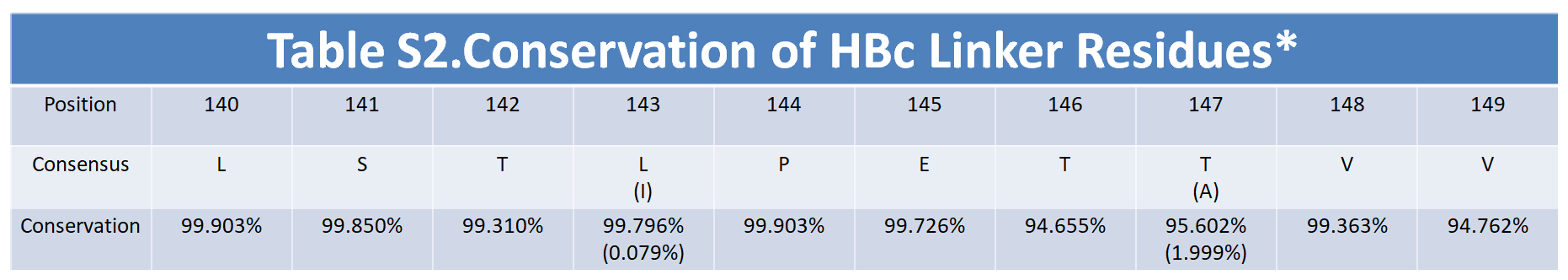


*****A total of 11,301 HBc sequences belonging to different HBV genotypes were downloaded from the HBVdb database and analyzed using the Clustal Omega sequence alignment program. The degree of conservation at the last position of the HBc NTD (140) and within the linker peptide (141-149) is shown. The frequency of I143 and A147, which were included in the current mutagenesis study, is shown in parentheses.
